# A systematic review with a Burden of Proof meta-analysis of health effects of long-term ambient fine particulate matter (PM_2.5_) exposure on dementia

**DOI:** 10.1038/s43587-025-00844-y

**Published:** 2025-03-21

**Authors:** Xinmei Huang, Jaimie Steinmetz, Elizabeth K. Marsh, Aleksandr Y. Aravkin, Charlie Ashbaugh, Christopher J. L. Murray, Fanghan Yang, John S. Ji, Peng Zheng, Reed J. D. Sorensen, Sarah Wozniak, Simon I. Hay, Susan A. McLaughlin, Vanessa Garcia, Michael Brauer, Katrin Burkart

**Affiliations:** 1 Institute for Health Metrics and Evaluation, University of Washington, Seattle, WA USA; 2 Department of Environmental and Occupational Health Sciences, School of Public Health, University of Washington, Seattle, WA USA; 3 Department of Health Metrics Sciences, School of Medicine, University of Washington, Seattle, WA USA; 4 School of Medicine, Washington University in St. Louis, St. Louis, MO USA; 5 Vanke School of Public Health, Tsinghua University, Beijing, China; 6 School of Population and Public Health, The University of British Columbia, Vancouver, British Columbia Canada

**Keywords:** Alzheimer's disease, Dementia, Ageing

## Abstract

Previous studies have indicated increased dementia risk associated with fine particulate matter (PM_2.5_) exposure; however, the findings are inconsistent. In this systematic review, we assessed the association between long-term PM_2.5_ exposure and dementia outcomes using the Burden of Proof meta-analytic framework, which relaxes log-linear assumptions to better characterize relative risk functions and quantify unexplained between-study heterogeneity (PROSPERO, ID CRD42023421869). Here we report a meta-analysis of 28 longitudinal cohort studies published up to June 2023 that investigated long-term PM_2.5_ exposure and dementia outcomes. We derived risk–outcome scores (ROSs), highly conservative measures of effect size and evidence strength, mapped onto a 1–5-star rating from ‘weak and/or inconsistent evidence’ to ‘very strong and/or consistent evidence’. We identified a significant nonlinear relationship between PM_2.5_ exposure and dementia, with a minimum 14% increased risk averaged across PM_2.5_ levels between 4.5 and 26.9 µg m^−3^ (the 15th to 85th percentile exposure range across included studies), relative to a reference of 2.0 µg m^−3^ (*n* = 49, ROS = 0.13, two stars). We found a significant association of PM_2.5_ with Alzheimer’s disease (*n* = 12, ROS = 0.32, three stars) but not with vascular dementia. Our findings highlight the potential impact of air pollution on brain aging.

## Abstract (web summary)

A systematic review with a Burden of Proof meta-analysis reporting a nonlinear relationship between ambient fine particulate matter and dementia across 28 longitudinal cohort studies, highlighting the potential impact of air pollution on brain aging.

## Main

Given the increase in the aging population worldwide^1^, dementia has emerged as a pressing public health issue. The 2021 Global Burden of Diseases, Injuries and Risk Factors Study (GBD) highlights dementia as the fifth leading cause of death and disability in 2021 among individuals aged 75 years and older, accounting for 1.66 million deaths (95% uncertainty interval (UI) 0.44–4.27), 36.4 million prevalent cases (30.9–42.7) and 25.3 million disability-adjusted life-years (11.7–53.6) globally^2,3^. With nearly 10 million new cases estimated each year, the World Health Organization (WHO) projects that the total number of people living with dementia will reach 152 million by 2050 (ref. ^4^).

Air pollution, especially fine particulate matter with a diameter ≤2.5 μm (PM_2.5_), has been identified as a potential risk factor for cognitive decline and dementia, with pathological and epidemiological studies supporting a plausible risk–outcome relationship^5–10^. With more than 90% of the global population exposed to annual PM_2.5_ concentrations exceeding the WHO air quality guideline of 5 μg m^−3^, the majority of individuals may face an elevated risk of dementia^11^. The Dementia Prevention, Intervention and Care report issued by the Lancet Commission in 2024 included air pollution as one of 14 modifiable risk factors for dementia, suggesting a weighted population attributable fraction (PAF; the proportional reduction in dementia risk that would occur if PM_2.5_ exposure were reduced to the theoretical minimum exposure level) of 2.6% (ref. ^12^); however, this estimated PAF was based on a single epidemiologic study and did not incorporate the spectrum of emerging evidence for effect estimates available in the recent literature.

Recent meta-analyses evaluating the epidemiological evidence of the PM_2.5_–dementia relationship indicate an adverse impact of elevated exposure levels, although with substantial variation in effect magnitudes across studies. Abolhasani et al. estimated a 3% (95% confidence interval (CI) 2–5) increase in dementia risk per 1 μg m^−3^ PM_2.5_ increase based on 14 studies^8^, while Cheng et al. reported a pooled hazard ratio (HR) of 1.03 (CI 1.02–1.05) for dementia per 1 μg m^−3^ increment of PM_2.5_ based on 18 studies^10^. Wilker et al. found an adverse but not significant impact of PM_2.5_ on dementia with an HR of 1.02 (CI 0.99–1.04) per 1 μg m^−3^ PM_2.5_ increase^13^. A few meta-analytical studies also attempted to evaluate the specific impact of PM_2.5_ on the two most common dementia subtypes of Alzheimer’s disease (AD) and vascular dementia (VaD). Cheng et al. reported a 4% (CI 2–6) elevated risk of AD per 1 μg m^−3^ increment of PM_2.5_ based on 13 studies^10^, while another meta-analysis found a nonsignificant impact of PM_2.5_ on AD across nine studies^14^. For VaD, one meta-analysis of eight cohort studies reported an HR of 1.07 (CI 1.03–1.12) per 1 μg m^−3^ PM_2.5_ (ref. ^10^).

However, existing meta-analytical literature on PM_2.5_ and dementia suffer from several limitations. Common meta-analytical frameworks, such as linear mixed-effect models, have been criticized as being overly simplistic, lacking appropriate bias assessment and unable to reflect the typically complex shape of risk–outcome relationships involving air pollution^15–17^. Furthermore, in traditional statistical approaches, between-study heterogeneity that is not explained by known differences in study-level characteristics is usually not adequately considered or quantitively assessed^16^.

In this study, we implemented the Burden of Proof framework recently developed by Zheng et al.^16,18^ to estimate the continuous dose–response relationship of ambient PM_2.5_ and dementia, including all types of dementia and specific subtypes such as AD and VaD. Notably, the method relaxes the conventional (log-)linear assumption for the relative risk (RR) curve, offering the flexibility required for optimal data fitting. It also provides a means to incorporate unexplained between-study heterogeneity into estimates of uncertainty after accounting for known variation in study design characteristics, in addition to accounting for small numbers of studies. The method also allows us to derive the Burden of Proof Risk Function (BPRF), which can be interpreted as the lowest, most conservative estimate of excess dementia risk associated with PM_2.5_ exposure, based on the available evidence. To facilitate interpretation and comparability, the BPRF, which incorporates both the strength of risk–outcome associations and the consistency of their underlying evidence, is translated into a risk–outcome score (ROS) and associated star rating, ranging from one star (weak and/or inconsistent evidence of an association) to five stars (very strong and/or consistent evidence of an association)^18^. The main findings and policy implications of this study are presented in Table 1.

**Table 1:** Policy summary

**Background**	Emerging pathological and epidemiological evidence indicates a connection between long-term ambient PM_2.5_ exposure and dementia risk. To date, most existing meta-regression studies rely on (log-)linear models, which are potentially insufficient to capture the true complexity of the PM_2.5_–dementia relationship. Moreover, these studies lack systematic consideration of unexplained between-study heterogeneity and assessment of evidence strength. In this meta-analysis, we applied the Burden of Proof methodology to synthesize evidence investigating the association between PM_2.5_ and dementia, including specific dementia subtypes, such as AD and VaD.
**Main findings and limitations**	Our results indicate a significant association between ambient PM_2.5_ exposure and dementia risk. Utilizing the conservative BPRF measure (the 5th percentile risk curve, inclusive of the uncertainty incorporating between-study heterogeneity, that is closest to the null) we found a minimum of 14% increase in dementia risk averaged across the 15th to 85th percentile exposure range (between 4.5 and 26.9 µg m^−3^), relative to the reference PM_2.5_ level of 2.0 µg m^−3^. In the BPRF framework, with one star suggesting weak and/or inconsistent evidence of association and five stars suggesting very strong and/or consistent evidence of association, the PM_2.5_–dementia relationship received a two-star rating. Our analysis yielded a nonlinear function indicating considerable increase in dementia risk as PM_2.5_ exposure rises to average levels (approximately 15 µg m^−3^), followed by a more modest increase in risk at higher PM_2.5_ levels. For dementia subtypes, a significant three-star association was found between ambient PM_2.5_ and AD, but no evidence for a relationship was observed for VaD outcomes. Limitations of this study are rooted in the current constraints inherent to the epidemiological evidence on PM_2.5_ and dementia, including the scarcity of published data on air pollution and subtype dementia, such as AD and VaD. Moreover, the available data reflect a geographical imbalance, with research primarily concentrated in North America, Europe and China, possibly limiting the generalizability of the estimated risk–outcome relationship. While the incorporation of bias covariates to systematically account for known variation in study design characteristics is an important advantage of the Burden of Proof approach, it is not possible to account for all potential confounders.
**Policy implications**	Our meta-analysis of existing evidence from epidemiological cohort studies reveals a concerning association between elevated ambient PM_2.5_ levels and an increased risk of dementia. Our findings underscore that even at low exposure levels (<15 µg m^−3^), dementia risk increases rapidly with increasing PM_2.5_ exposure. Consequently, it is imperative for policymakers globally to align national PM_2.5_ standards with WHO guidelines (5 µg m^−3^). Simultaneously, it is crucial for policymakers to support monitoring of ambient PM_2.5_ concentrations to uphold established air quality benchmarks. Collaborative efforts involving governmental bodies and healthcare professionals are essential to enhance public awareness of the adverse effects of air pollution on brain health and to promote healthy aging practices. These approaches hold the potential to dramatically mitigate the risk of dementia and enhance overall public health and well-being.

## Results

### Overview

**Extended Data Fig. 1:**
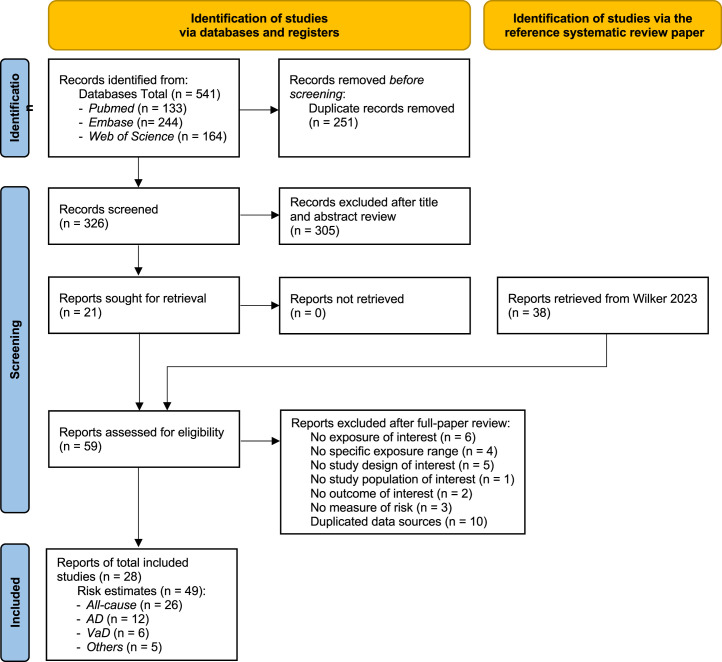
PRISMA flow diagram of PM_2.5_ exposure and dementia outcomes. Template is from Page MJ, McKenzie JE, Bossuyt PM, Boutron I, Hoffmann TC, Mulrow CD, et al. The PRISMA 2020 statement: an updated guideline for reporting systematic reviews. BMJ 2021;372:n71. 10.1136/bmj.n71. For more information, visit: http://www.prisma-statement.org/.

We systematically searched the literature for studies assessing the impact of long-term PM_2.5_ exposure on dementia risk, following the Preferred Reporting Items for Systematic Reviews and Meta-Analyses (PRISMA) guidelines^19^. We initially identified the most recent peer-reviewed systematic review that complied with PRISMA guidelines, namely, a study by Wilker et al.^13^ that spanned the periods from the inception of each database searched to July 2022. To generate an updated systematic review, we employed search strings in alignment with those used in Wilker et al.^13^ and searched PubMed, Embase and Web of Science to further identify studies published between July 2022 and June 2023 (Extended Data Fig. 1). Our search initially identified 38 papers reviewed in Wilker et al.^13^, which we supplemented with an additional 21 papers retrieved from the database search. After evaluation, we excluded 31 papers that did not meet our inclusion criteria. (The search terms and inclusion/exclusion criteria are outlined in the Supplementary Information and the detailed inclusion/exclusion decisions and rationale for each study are provided in Supplementary Table 4). In total, we integrated data from 28 longitudinal cohort studies^20–47^, which contributed a collective total of 49 HRs to our meta-regression analysis. More specifically, we incorporated 26 HRs^21–28,30,31,33–40,42,43,45–47^ for all-cause dementia outcomes, 12 HRs^20,22,25,28,30–32,34,40,41,43,44^ for AD outcomes, six HRs^22,24,25,28,40,43^ for VaD outcomes and five HRs^22,25,29–31^ for other types of dementia (Extended Data Fig. 1).

**Extended Data Fig. 2:**
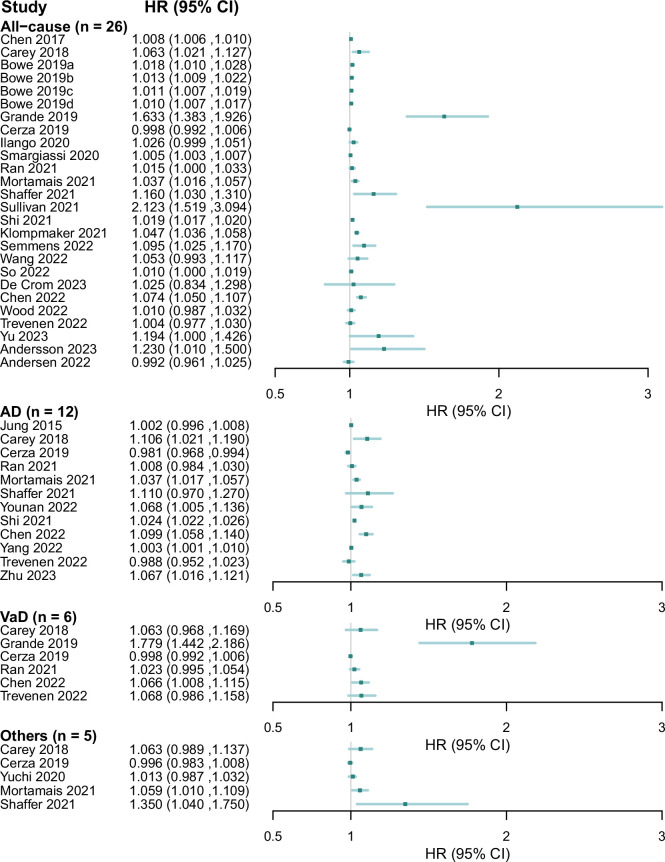
Forest plot of the extracted Hazard Ratios (HR) and corresponding 95% confidence intervals (CI) from all included studies for each dementia outcome, including all-cause dementia (n = 26), Alzheimer’s disease (AD) (n = 12), vascular dementia (VaD) (n = 6), and other types of dementia (n = 5). The reported HR from the most-adjusted model was extracted for each dementia outcome from each study. If multiple averaging periods were implemented in the same study, the effect estimate using the longest averaging period were extracted and used as input for the meta-analyses. For Bowe 2019, four effect estimates were extracted from nonlinear risk–outcome curve at PM_2.5_ exposure level of a. 6 μg/m^3^, b. 10 μg/m^3^, c. 13 μg/m^3^, and 16 μg/m^3^, representing the exposure range of this study. All study-specific effect estimates were scaled to a standard unit change in PM_2.5_ concentration of 1 μg/m^3^. Dark green squares represent mean HRs, and light green lines indicate 95% CIs from each study. See Supplementary Table 4 for more details on included observations from each study.

Collectively, the studies included in our meta-analysis comprised 11 cohort studies conducted in Europe, nine in the United States, four in China, three in Canada and one in Australia (Supplementary Table 5). The aggregated data included approximately 2.73 million incident dementia cases and dementia mortalities, which were derived from a total population of 36.3 million individuals. The duration of follow-up varied among the studies, ranging from 3 to 25 years, with a median of 13 years (interquartile range (IQR) 9–15 years). Estimated long-term ambient PM_2.5_ exposure levels exhibited considerable variability, ranging from 0.1 to 61.7 µg m^−3^ PM_2.5_ across the entire participant pool. Detailed study characteristics are in Supplementary Table 5. Extended Data Fig. 2 provides the extracted HR from each included study for all-cause dementia, AD, VaD and other types of dementia, all scaled for a standard PM_2.5_ increment of 1 µg m^−3^. The meta-analytic results, including effect estimates, key analytic parameters and characteristics of the PM_2.5_–dementia relationship, in addition to results from analysis restricted to dementia subtypes (AD and VaD), are presented in Table 2.

**Table 2:** Strength of the evidence for the relationship between exposure to ambient PM_2.5_ and dementia outcomes analyzed

Health outcome	RR for PM_2.5_ level of 15.5 μg m^−3^ (95% UI without between-study heterogeneity)	RR for PM_2.5_ level of 15.5 μg m^−3^ (95% UI with between-study heterogeneity)	BPRF	ROS	Star rating	Publication bias	No. of effect estimates	Selected bias covariates	Risk–outcome pair in GBD 2023
All dementia outcomes combined	1.37 (1.20–1.57)	1.37 (1.13–1.69)	1.14	0.13	☆☆	Yes	49	Covariate for AD (1, AD; 0, other dementia types other than AD)	Yes
Subtype: AD	1.39 (1.35–1.43)	1.39 (1.33–1.44)	1.38	0.32	☆☆☆	Yes	12	None	No
Subtype: VaD	3.15 (0.91–10.65)	3.13 (0.81–13.48)	–	–	–	No	6	Covariate for the selection bias (0, low risk; 1, medium risk; 2, high risk)	No

The reported mean RR and its 95% UI reflect the risk an individual who has been exposed to the mean PM_2.5_ exposure level across all included studies (15.5 μg m^−3^) has of developing the outcome of interest relative to that of someone who has not been exposed to the reference PM_2.5_ level of 2.0 μg m^−3^. The health outcome ‘Dementia’ includes extracted effect estimates from all included studies, whereas AD and VaD only include effect estimates for the corresponding dementia subtypes. We report the 95% UI when not incorporating between-study heterogeneity and when accounting for between-study heterogeneity. The BPRF is calculated for risk–outcome pairs that were found to have significant relationships at a 0.05 level of significance when not incorporating between-study heterogeneity (the lower bound of the 95% UI without γ does not cross the null RR value of one). The BPRF corresponds to the fifth quantile estimate of RR accounting for between-study heterogeneity closest to the null for each risk–outcome pair, and it reflects the most conservative estimate of excess risk associated with ambient PM_2.5_ exposure that is consistent with the available data. The ROS is calculated as log(BPRF). Negative ROSs indicate that the evidence of the association is very weak and inconsistent. For ease of interpretation, we have transformed the ROS and BPRF into a star rating (1–5) with a higher rating representing a larger effect with stronger evidence. The potential existence of publication bias, which, if present, would affect the validity of the results, was tested using Egger’s regression. Included studies represent all available relevant data identified through our systematic reviews up to June 2023. The selected bias covariates were chosen for inclusion in the model using algorithm that systematically detects bias covariates that correspond to significant sources of bias in the observations included. If selected, the observations were adjusted to better reflect the gold-standard values of the covariate. The Supplementary Information provides more information about the candidate bias covariates that were selected for in each model. The risk–outcome pairs in this study were not included in GBD 2021 analyses but are planned for inclusion in the next iteration of GBD.

### PM_2.5_ and dementia

**Fig. 1:**
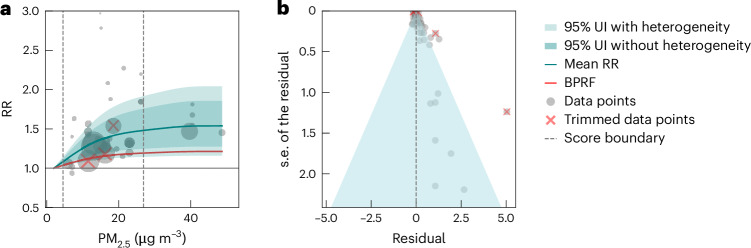
Relationships between PM_2.5_ exposure and dementia (49 data points from 28 studies). **a**, RR function. The solid green line represents the mean RR at each exposure level, and the solid red line represents the BPRF. The dark green shaded area indicates the 95% UI without accounting for between-study heterogeneity, and the light green shaded area represents the 95% UI accounting for between-study heterogeneity. The size of the data points is proportional to the inverse of the s.d. of the effect estimates, where larger points indicate higher precision in the effect estimates. **b**, A modified funnel plot showing the residuals (relative to zero) on the *x* axis and the estimated s.d. (inclusive of between-study heterogeneity) on the *y* axis.

Our main meta-regression—Bayesian, regularized, trimmed (MR-BRT) model (Methods) for all dementia outcomes combined revealed a statistically significant relationship between ambient PM_2.5_ exposure and dementia risk. We found a consistent increase in the risk of dementia incidence and mortality across the exposure range (Fig. 1 and Supplementary Table 7). Notably, our analysis delineated a monotonically increasing curve characterizing this association, indicating a considerable rise in risk until PM_2.5_ levels reached approximately 15 µg m^−3^, followed by a more modest risk increase at higher PM_2.5_ levels. At the observed mean PM_2.5_ exposure level of 15.5 µg m^−3^ across all included studies, the mean RR of dementia was 1.37 (95% UI inclusive of between-study heterogeneity 1.13–1.69), compared to the reference PM_2.5_ exposure of 2.0 µg m^−3^ (Table 2). The reference PM_2.5_ exposure in our study represents the lowest observed reference level across all the included studies.

In our conservative BPRF-based assessment of the evidence, which complements conventional RR estimates, the BPRF is defined for harmful risks as the fifth percentile estimated risk curve inclusive of between-study heterogeneity that is closest to the null^16,18^, illustrated by the red line in Fig. 1a. The exposure-averaged BPRF of 1.14 demonstrates that ambient PM_2.5_ exposure in the range of the 15th percentile to 85th percentile (PM_2.5_, 4.5–26.9 µg m^−3^) elevates the risk of dementia by at least 14% on average, relative to a reference value of 2.0 µg m^−3^. The estimated harmful association between PM_2.5_ and dementia, as indicated by the corresponding ROS (the signed value of the log exposure-averaged BPRF) of 0.13, corresponds to a two-star rating (Table 2).

### Accounting for study bias and quantifying heterogeneity

Among the 12 candidate covariates identified based on six primary sources of bias, per Grading of Recommendations, Assessment, Development and Evaluations (GRADE) criteria^48^, including representativeness of the study population, exposure measurement, outcome assessment, potential for reverse causation, control for confounding variables and selection bias (step 3 of Methods and Supplementary Table 6), only the covariate representing the AD outcome (1 for AD outcomes, 0 for non-AD outcomes) was found to have a significant effect on the RR curve and therefore included as a covariate in the final model for all dementia outcomes combined. In a separate step, we used a Fisher information matrix^49^ to quantify any remaining unexplained heterogeneity and integrated this estimate into uncertainty intervals. Our analysis revealed a sustained level of moderate unexplained between-study heterogeneity within our set of included studies, evident from the 95% UI inclusive of between-study heterogeneity for the RR curve, as illustrated in light blue shades in Fig. 1a.

Our analysis additionally uncovered evidence of publication bias, supported by a significant *P* value (0.03) derived from the Egger’s regression test^50^. The asymmetry evident in the funnel plot (Fig. 1b) illustrates a skewed distribution, notably with a clustering of data predominantly on the right side, where the residual is larger than zero. This imbalance suggests a propensity for studies with notably positive results to be more prominently featured within the published literature. The Supplementary Information contains detailed results for our assessment of study quality and risk of bias.

### Subgroup analysis

**Fig. 2:**
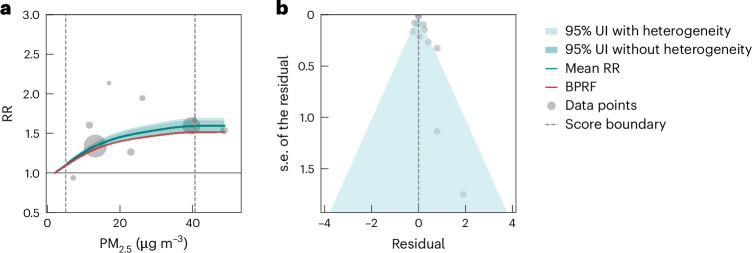
Relationships between PM_2.5_ exposure and Alzheimer’s disease (12 data points from 12 studies). **a**, RR function. The solid green line represents the mean RR at each exposure level, and the solid red line represents the BPRF. The dark green shaded area indicates the 95% UI without accounting for between-study heterogeneity, while the light green shaded area represents the 95% UI accounting for between-study heterogeneity. The size of the data points is proportional to the inverse of the s.d. of the effect estimates, where larger points indicate higher precision in the effect estimates. **b**, A modified funnel plot showing the residuals (relative to zero) on the *x* axis and the estimated s.d. (inclusive of between-study heterogeneity) on the *y* axis.

**Fig. 3:**
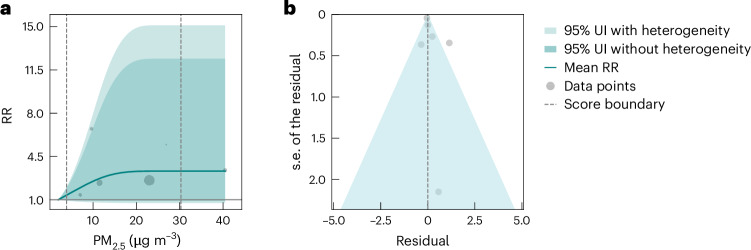
Relationships between PM_2.5_ exposure and vascular dementia (six data points from six studies). **a**, RR function. The solid green line represents the mean RR at each exposure level. The dark green shaded area indicates the 95% UI without accounting for between-study heterogeneity, while the light green shaded area represents the 95% UI accounting for between-study heterogeneity. The size of the data points is proportional to the inverse of the s.d. of the effect estimates, where larger points indicate higher precision in the effect estimates. **b**, A modified funnel plot showing the residuals (relative to zero) on the *x* axis and the estimated s.d. (inclusive of between-study heterogeneity) on the *y* axis.

We conducted subgroup analyses specifically focusing on the association between PM_2.5_ exposure and AD and VaD outcomes, depicted by the risk curves in Figs. 2a and 3a, respectively. We included 12 studies for AD and six studies for VaD and (unlike the approach we took for the full dementia analysis) did not trim the data for outliers due to the limited datasets for studies for dementia subtypes (Extended Data Fig. 1 and Supplementary Table 5). Our findings indicated a significant association between elevated PM_2.5_ exposure and the risk of AD (Fig. 2a). At the mean exposure level of 15.5 µg m^−3^, the mean RR for AD was estimated to be 1.39 (95% UI inclusive of between-study heterogeneity 1.33–1.44) compared to the reference PM_2.5_ level (Table 2). It is noteworthy that even when including between-study heterogeneity, the 95% UI remained remarkably narrow. On average, as indicated by an estimated BPRF of 1.38, AD increased by at least 38% for populations exposed to PM_2.5_ levels between the 15th and 85th percentile (5.0–40.7 µg m^−3^) as compared to those exposed to a reference level of 2.0 µg m^−3^. The ROS for PM_2.5_ exposure and AD outcomes was 0.32, representing a three-star rating risk–outcome pair. Conversely, while the mean VaD risk tended to rise with increased PM_2.5_ exposure, our analysis did not find a significant association (Fig. 3a and Table 2). The notably wide 95% UI indicated that the PM_2.5_–VaD relationship was characterized by a high degree of uncertainty and sensitivity. For detailed estimates of risks of both AD and VaD in response to PM_2.5_ exposure, including their corresponding 95% UI inclusive of heterogeneity, refer to Supplementary Tables 8 and 9. We observed significant publication bias for AD (*P* = 0.04) but not for VaD.

### Stratified and sensitivity analyses

**Extended Data Fig. 3:**
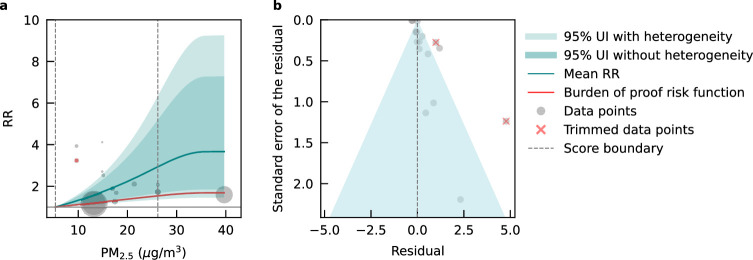
Relationships between PM_2.5_ exposure and dementia among studies with active ascertainment through physician diagnosis (17 data points from 10 studies). **a**, RR function. The solid green line represents the mean RR at each exposure level, and the solid red line represents the Burden of proof risk function. The dark green shaded area indicates the 95% uncertainty interval (UI) without accounting for between-study heterogeneity, while the light green shaded area represents the 95% UI accounting for between-study heterogeneity. The size of the data points is proportional to the inverse of the standard deviation of the effect estimates, where larger points indicate higher precision in the effect estimates. **b**, A modified funnel plot showing the residuals (relative to zero) on the x axis and the estimated s.d. (inclusive of between-study heterogeneity) on the y axis.

**Extended Data Fig. 4:**
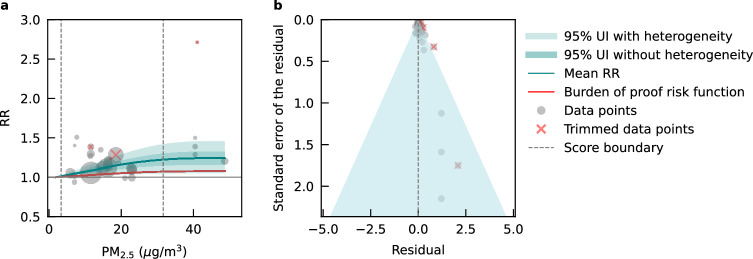
Relationships between PM_2.5_ exposure and dementia among studies with passive ascertainment through medical records, death certificates, or self-reports (32 data points from 18 studies). **a**, RR function. The solid green line represents the mean RR at each exposure level, and the solid red line represents the Burden of proof risk function. The dark green shaded area indicates the 95% uncertainty interval (UI) without accounting for between-study heterogeneity, while the light green shaded area represents the 95% UI accounting for between-study heterogeneity. The size of the data points is proportional to the inverse of the standard deviation of the effect estimates, where larger points indicate higher precision in the effect estimates. **b**, A modified funnel plot showing the residuals (relative to zero) on the x axis and the estimated s.d. (inclusive of between-study heterogeneity) on the y axis.

**Extended Data Fig. 5:**
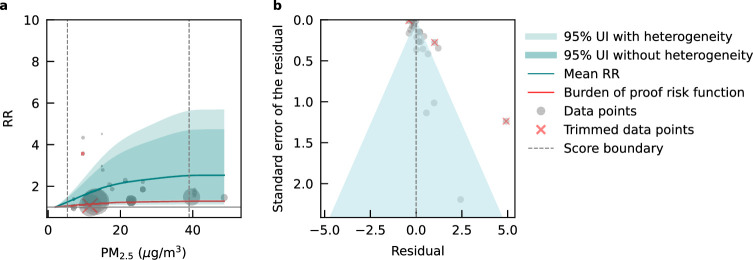
Relationships between PM_2.5_ exposure and dementia among studies restricted to participants aged ≥ 60 years at baseline (28 data points from 15 studies). **a**, RR function. The solid green line represents the mean RR at each exposure level, and the solid red line represents the Burden of proof risk function. The dark green shaded area indicates the 95% uncertainty interval (UI) without accounting for between-study heterogeneity, while the light green shaded area represents the 95% UI accounting for between-study heterogeneity. The size of the data points is proportional to the inverse of the standard deviation of the effect estimates, where larger points indicate higher precision in the effect estimates. **b**, A modified funnel plot showing the residuals (relative to zero) on the x axis and the estimated s.d. (inclusive of between-study heterogeneity) on the y axis.

**Extended Data Fig. 6:**
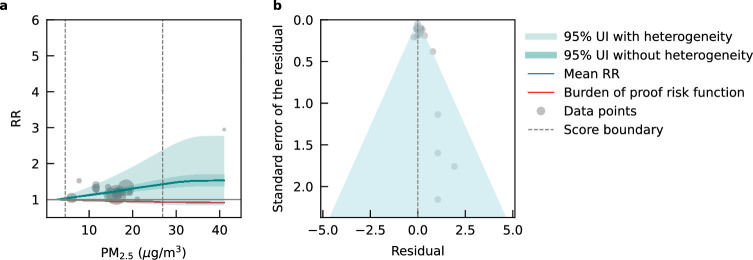
Relationships between PM_2.5_ exposure and dementia among studies with a broader baseline age range, not limited to ≥ 60 years (21 data points from 13 studies). **a**, RR function. The solid green line represents the mean RR at each exposure level, and the solid red line represents the Burden of proof risk function. The dark green shaded area indicates the 95% uncertainty interval (UI) without accounting for between-study heterogeneity, while the light green shaded area represents the 95% UI accounting for between-study heterogeneity. The size of the data points is proportional to the inverse of the standard deviation of the effect estimates, where larger points indicate higher precision in the effect estimates. **b**, A modified funnel plot showing the residuals (relative to zero) on the x axis and the estimated s.d. (inclusive of between-study heterogeneity) on the y axis. No trimming was performed to maintain model convergence.

**Extended Data Fig. 7:**
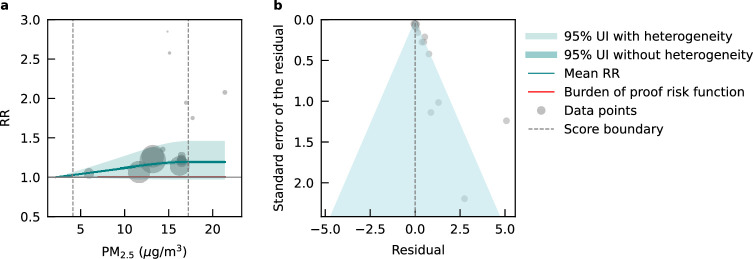
Relationships between PM_2.5_ exposure and dementia among studies conducted in North America (18 data points from 12 studies). **a**, RR function. The solid green line represents the mean RR at each exposure level, and the solid red line represents the Burden of proof risk function. The dark green shaded area indicates the 95% uncertainty interval (UI) without accounting for between-study heterogeneity, while the light green shaded area represents the 95% UI accounting for between-study heterogeneity. The size of the data points is proportional to the inverse of the standard deviation of the effect estimates, where larger points indicate higher precision in the effect estimates. **b**, A modified funnel plot showing the residuals (relative to zero) on the x axis and the estimated s.d. (inclusive of between-study heterogeneity) on the y axis. No trimming was performed to maintain model convergence.

**Extended Data Fig. 8:**
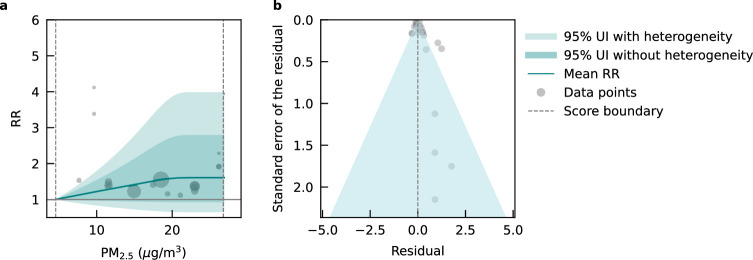
Relationships between PM_2.5_ exposure and dementia among studies conducted in Europe (22 data points from 11 studies). **a**, RR function. The solid green line represents the mean RR at each exposure level, and the solid red line represents the Burden of proof risk function. The dark green shaded area indicates the 95% uncertainty interval (UI) without accounting for between-study heterogeneity, while the light green shaded area represents the 95% UI accounting for between-study heterogeneity. The size of the data points is proportional to the inverse of the standard deviation of the effect estimates, where larger points indicate higher precision in the effect estimates. **b**, A modified funnel plot showing the residuals (relative to zero) on the x axis and the estimated s.d. (inclusive of between-study heterogeneity) on the y axis. No trimming was performed to maintain model convergence.

We conducted three sensitivity analyses to examine variations in potential PM_2.5_–dementia associations. First, stratified analysis by dementia case diagnosis revealed stronger evidence for an association in the ten included studies using active ascertainment of dementia cases (ROS = 0.23, three-star) compared to those (18 studies) relying on passive diagnosis or self-reported cases (ROS = 0.04, two-star) (Extended Data Figs. 3 and 4). Second, in a subset of 15 studies restricted to older adults with a baseline age ≥60 years, we observed a three-star association (ROS = 0.19), showing stronger evidence compared to the 13 included studies including participants with a broader baseline age range (ROS < 0, one star) (Extended Data Figs. 5 and 6). Third, subregion analyses indicated a two-star association (ROS = 0.001) among 12 studies conducted in North America, whereas no consistent evidence was found for 11 studies conducted in Europe (Extended Data Figs. 7 and 8). Due to limited data availability, subregion analyses were not performed for other regions. Notably, the stratified analyses for studies using active ascertainment and for the older adults both showed stronger evidence compared to the overall association observed in the main analysis (ROS = 0.13, two-star).

To test the model fitting and robustness, we further implemented two alternative models without trimming and with 15% trimming. Comparing the mean risk curves estimated from these two models to the main model for all dementia outcomes combined that employed 10% trimming, no significant differences were found (Supplementary Fig. 1). Even when including outliers without trimming, the estimated association between ambient PM_2.5_ and dementia risk remained significant. However, a noticeably wider 95% UI inclusive of between-study heterogeneity was evident. Both alternative models retained a two-star rating, but the estimated ROS was lower compared to the main model, with an ROS of 0.14 for the 15%-trimming model and 0.08 for the no-trimming model. Furthermore, we conducted sensitivity analyses to investigate the influence of adjusting the monotonicity constraint and utilizing cubic splines on the estimated risk curves. Notably, the removal of enforced model shape constraints did not yield substantial alterations in the risk curve outcomes for all-type dementia and AD (Supplementary Fig. 1 and Figs. 2 and 3). However, the risk curves related to VaD demonstrated pronounced sensitivity to these specific constraints (Supplementary Fig. 1 and Figs. 2 and 3).

## Discussion

Our study systematically reviewed 28 cohort studies providing 49 effect estimates characterizing the relationship between long-term ambient PM_2.5_ exposure and dementia. Using the Burden of Proof meta-analytical framework, we constructed flexible RR functions that did not presume a (log-)linear relationship, demonstrating a significant association between increased PM_2.5_ exposure levels and heightened risks of all dementia outcomes combined. The estimated RR curve indicates a notably steeper rise in dementia risk at lower PM_2.5_ levels before reaching the mean exposure level across all included studies, approximately 15 µg m^−3^. Beyond this point, the rise in RR with increasing PM_2.5_ exposure becomes much less steep. Based on the conservative BPRF metrics our method generates in conjunction with the RR function, we estimated a minimum average increase in dementia risk of 14% associated with ambient PM_2.5_ exposure between 4.5 and 26.9 µg m^−3^ (relative to a reference level of 2.0 µg m^−3^). The two-star rating, derived from an ROS of 0.13, is interpreted in the Burden of Proof framework as indicating weak evidence of an association, based on the available data.

Our subtype analysis highlights that PM_2.5_ exposure may impact dementia risk differently across subtypes. AD, as represented by a binary covariate, proved to be significant in the overall PM_2.5_–dementia analysis; it was the only significant covariate identified by our algorithm from a set of 12 covariates. When conducting subtype-specific meta-regression, our conservative BPRF metrics indicated a significant relationship between PM_2.5_ and AD, yielding a three-star rating (ROS = 0.32), indicating a moderate association and/or moderately consistent evidence given the available data. Our study demonstrated stronger evidence for the PM_2.5_–AD relationship compared to the association between PM_2.5_ and all dementia outcomes combined (two stars). Notably, the 95% UI around the RR function for AD is very narrow, even when incorporating unexplained between-study heterogeneity, which at least partially explains the finding that the evidence for the PM_2.5_–AD relationship is relatively strong. Meanwhile, based on a limited number of studies (*n* = 6), we did not observe a significant relationship between VaD and PM_2.5_ exposure, however, the mean effect of PM_2.5_ suggests there may be an increase in VaD risk. Figure 2a illustrates the considerable heterogeneity in effect estimates for VaD that likely underlie the large uncertainty estimates. This heterogeneity is partly driven by one study, which reported a significantly higher effect estimate with greater uncertainty, based on an analysis with only 70 VaD cases among 2,927 participants^24^. With VaD comprising 10–20% of dementia cases compared to 40–60% for AD^51,52^, its lower incidence likely reduces the power of the statistical analysis and amplifies the uncertainty in the results. Another major source of heterogeneity stems from the clinical diagnosis of dementia subtypes. It is common that information regarding dementia subtype diagnosis is inaccurate or missing, particularly when relying on passive ascertainment, leading to potential under-reporting and misclassification^53,54^; most of the subtype studies included (7 out of 12 for AD, and 5 out of 6 for VaD) used administrative medical records or death certificates. Furthermore, as observed in recent neuropathological studies^55,56^, AD, VaD, and other dementia subtypes may display a co-occurrence of multiple neuropathological hallmarks, indicating that current clinical diagnoses of dementia subtypes are an imperfect science and may not always reflect all underlying brain pathologies accurately. Possible misclassification or over-classifications may have introduced potential biases into our subgroup analyses.

The findings from our stratified analyses highlight the influence of dementia diagnosis methods, population characteristics, and regional differences on the estimated PM_2.5_–dementia associations. Stratified analysis by diagnosis methods revealed stronger evidence for an association in studies using active ascertainment through physician diagnosis (ROS = 0.23, three stars) compared to those relying on passive ascertainment via medical records, death certificates or self-reports (ROS = 0.04, two stars). These results align with a previous meta-analysis of PM_2.5_ and dementia, which reported significant associations only in studies employing active ascertainment, with no significant findings in studies using passive ascertainment methods^13^. The weaker associations observed in studies with passive ascertainment or self-reports are likely biased toward the null due to a higher risk of outcome misclassification or underdiagnosis in these methods compared to active ascertainment^57^. Analysis restricted to studies involving older adults (baseline age ≥60 years) revealed stronger evidence for the association between PM_2.5_ exposure and dementia (ROS = 0.19, three stars), compared to the main analysis including all studies (ROS = 0.13, two stars). The higher mean RR observed among the older adults focused studies suggests that older individuals may be more vulnerable to PM_2.5_ exposure, potentially due to increased susceptibility linked to age-related declines in physiological resilience and greater pre-existing health risks, amplifying PM_2.5_-related dementia risk^58^. Subregional analyses showed weaker evidence for PM_2.5_–dementia associations in studies conducted in North America (ROS = 0.001, one star) and no significant evidence in Europe, compared to the main analysis. These regional variations may reflect differences in PM_2.5_ sources, composition and exposure levels^59,60^, as well as population characteristics, socioeconomic factors and variability in dementia diagnostic methods across regions^61^.

Our study findings corroborate the positive association between PM_2.5_ exposure and dementia, which is supported by neuropathological studies demonstrating a biologically plausible effect of ambient PM_2.5_ on dementia risk^5–7^. PM_2.5_, particularly nanoscaled particles, can penetrate the human brain via two primary pathways: a direct route through the olfactory nerve, and an indirect route through the circulation system that crosses the blood–brain barrier^6,62–65^. Once entering the brain, PM_2.5_ has the potential to increase neurological oxidative stress, neuroinflammation, neurotoxicity, neurovascular damage and impairment of the blood–brain barrier and brain cells^5–7,66–69^. Through the systemic pathway, PM_2.5_ may even cause systemic oxidative stress and inflammation, thereby contributing to the progression of dementia^62,70^. In addition, epidemiological and clinical evidence has linked PM_2.5_ exposure to heightened cardiovascular disease risks, including conditions like hypertension and atherosclerosis^71,72^. These cardiovascular conditions may adversely impact cerebral blood flow and compromise neurovascular function, potentially elevating the risk of developing dementia^73^. Moreover, in the context of AD risk, other potential mechanisms involving PM-induced modifications in AD-related genes through DNA methylation, coupled with an elevated risk of brain amyloid-β deposition and tau protein neurofibrillary tangles, have been suggested in the literature^74,75^. Collectively, these factors contribute to a heightened risk of neurodegeneration, cognitive decline and the onset of dementia.

Our study has several key advantages over previous meta-analyses evaluating the relationship between PM_2.5_ exposure and dementia risk. First, it incorporated a greater number of individual studies and effect estimates than did previous meta-analyses. Second, the Burden of Proof analysis framework relaxes the (log-)linear assumptions common to traditional random effects models, allowing the implementation of flexible splines with monotonicity constraints. Moreover, our methodology involves a robust process for identifying and trimming outliers within the dataset to mitigate the impact of the inconsistency from existing literature. Notably, our conservative BPRF metrics incorporate unexplained between-study heterogeneity remaining after testing predefined bias covariates from a candidate set consistent with GRADE^48^ and Cochrane^76^ criteria and including significant covariates in the model, thus reducing the risk of spurious significant associations.

Additionally, our analysis provides an ROS and overarching star rating for the PM_2.5_–dementia relationships examined, facilitating a more intuitive assessment of the strength of evidence and comparison with other Burden of Proof evaluations of risk–outcome relationships involving dementia or air pollution. In comparison to other risk factors evaluated for associations with dementia included in recent GBD studies, namely, smoking (ROS = 0.09, two stars), high fasting plasma glucose (ROS = 0.04, two stars), and high body mass index (ROS = −0.07, one star), we estimated a higher ROS for PM_2.5_–dementia (ROS = 0.13, two stars)^77,78^. Additionally, when considering PM_2.5_ exposure-related risk–outcome relationships investigated in the current GBD analyses, the PM_2.5_–dementia association is weaker than that for PM_2.5_ and chronic obstructive pulmonary disease (ROS = 0.44, four stars); tracheal, bronchus and lung cancer (ROS = 0.34, three stars); ischemic heart disease (ROS = 0.26, three stars); diabetes mellitus type 2 (ROS = 0.19, three stars); stroke (ROS = 0.17, three stars); and lower respiratory infections (ROS = 0.15, three stars)^77^. The ROSs and star ratings for all risk–outcome pairs analyzed using the Burden of Proof framework and BPRF metrics are further accessible online through the Burden of Proof online visualization tool (https://vizhub.healthdata.org/burden-of-proof/).

Our ability to estimate a nonlinear dose–response function, as afforded by our flexible Burden of Proof methods, revealed that even at relatively low exposure levels (<15 µg m^−3^), there is a rapidly increasing risk of dementia with increasing ambient PM_2.5_ exposure. This finding underscores the urgency and necessity for policymakers to adjust air quality regulations to reduce air pollution levels that can negatively impact human brain health and healthy aging. The current WHO air quality guideline (AQG) recommends that the annual PM_2.5_ exposure concentration should not exceed 5 µg m^−3^ (ref. ^79^); however, existing national or regional air quality policies with legal enforcement are generally less stringent than the WHO AQG. For instance, the US National Ambient Air Quality Standards set the annual mean PM_2.5_ limit at 9 µg m^−3^ (ref. ^80^); Australia’s standard is 8 µg m^−3^ (ref. ^81^); and Canada’s is 8.8 µg m^−3^ (ref. ^82^). The United Kingdom aims to meet a 10 µg m^−3^ standard by 2040 (ref. ^83^). The European Union’s stage-two standard is currently 20 µg m^−3^ (ref. ^84^); however, a provisional political agreement was reached in February 2024 to lower this to 10 µg m^−3^ by 2030 (ref. ^85^). Japan’s standard is 15 µg m^−3^ (ref. ^86^), while China allows up to 35 µg m^−3^ (ref. ^87^). Given that more than 90% of people live in areas where PM_2.5_ levels exceed the WHO AQG guideline^11^, there is a pressing need for updated policies and adequate enforcement of air pollution regulations to reduce ambient PM_2.5_ exposure globally. Our findings can help raise public awareness about the adverse impacts of air pollution on dementia risk, thereby supporting the development and adoption of stricter air quality regulations. Effective policy adjustments are crucial for mitigating the health risks associated with PM_2.5_ exposure and safeguarding public health, particularly in terms of human brain health outcomes.

Our study is constrained by several limitations related to both input data and the modeling approach. Concerning input data, our meta-analysis is limited by the small number of published studies; in particular, constraining our subtype analysis, which included 12 studies for AD and 6 studies for VaD. Additionally, we were unable to perform meta-analyses for other dementia subtypes, such as senile dementia, frontotemporal dementia and Lewy body dementia, due to the scarcity or absence of relevant longitudinal epidemiological studies. Furthermore, the dataset displays a geographical imbalance, with input studies heavily concentrated in North America, Europe and Asia, potentially limiting global representation. Moreover, our analysis focuses exclusively on ambient PM_2.5_ exposure, without assessing the potential impacts of other air pollutants, such as O_3_ and NO_2_. Nor did we explore the potential combined effects of multiple pollutants on dementia risk due to the limited data availability, with only four studies accounting for PM_2.5_ and O_3_ combined effects^20,34,38,47^ and four on PM_2.5_ and NO_2_ combined effects^27,34,38,47^. Future research on both the individual effects of other pollutants besides PM_2.5_ on dementia risk as well as their joint impacts to provide a more comprehensive understanding of how air pollution contributes to dementia. In terms of the modeling approach, it is possible that our framework, despite the application of rigorous statistical methods, may not comprehensively address all potential sources of heterogeneity and bias. Although we attempted to account for known variation in study characteristics through comprehensive bias covariates, such bias might not be captured and corrected in the final model if all or the majority of the input studies are biased.

In conclusion, applying the Burden of Proof framework of Zheng et al.^16^, our systematic meta-analysis found a significant association between ambient PM_2.5_ exposure and dementia, deriving nonlinear RR curves from 28 epidemiological cohort studies available up to June 2023. Despite a moderate degree of between-study heterogeneity within the existing evidence, our synthesis revealed a weak association between PM_2.5_ exposure and dementia. For subtype analyses, a stronger association emerged between ambient PM_2.5_ and AD outcomes supported by moderate evidence (three stars), whereas no consistent evidence of an association across reviewed studies was found for VaD. Our meta-analytic assessments of the impact of long-term exposure to ambient PM_2.5_ on dementia yield useful data for stakeholders including policymakers, public health professionals and the general public. These insights can be used to inform policy formation and advance the protection and enhancement of human brain health through improved air pollution management.

## Methods

### Overview

This meta-analysis is based on the recently developed ‘Burden of Proof’ framework published by Zheng et al.^16,18^, and the implementations are disseminated using open-source Python libraries^88,89^. We used the MR-BRT model, a meta-analytic tool utilizing Bayesian regularized splines, to estimate the dose–response risk curves for long-term ambient PM_2.5_ exposure and dementia, relaxing the assumption of a log(linear) relationship. In addition, we conservatively estimated the BPRF for the risk–outcome association incorporating between-study heterogeneity to evaluate the strength of evidence across the included studies supporting the association. The ‘Burden of Proof’ framework has been employed to assess health effects associated with other risk factors^90–97^. This meta-analytic methodology consists of six main steps: (1) conduct a systematic review and extract data from identified studies reporting on the association between long-term ambient PM_2.5_ exposure and dementia using a GBD-standardized systematic review protocol; (2) estimate the continuous risk–outcome relationship, using the minimum exposure level among all included studies as the reference; (3) test and adjust for systematic biases as a function of known input study attributes; (4) quantify remaining unexplained between-study heterogeneity while adjusting for within-study correlation induced by computing the RRs for several alternatives with the same reference, as well as the number of studies; (5) assess evidence for small-study effects to evaluate potential risk of publication or reporting bias; and (6) estimate the BPRF, which provides a conservative measure of the average risk increase across the range of exposure supported by the evidence and is to compute the ROS, and subsequently map the ROS into five categories of star ratings.

The estimates for our primary indicators from this work (RRs across a range of exposures, BPRFs, ROSs and star ratings for ambient PM_2.5_ and dementia outcomes) are not specific to or disaggregated by specific populations (we did not estimate by location, sex or age group; and this analysis evaluated the effects of long-term exposure to PM_2.5_ on dementia in adults 30 years and older only); however, we did include studies that were conducted in female-only populations.

We followed PRISMA guidelines^19^ (Supplementary Tables 10 and 11 and Extended Data Fig. 1) and Guidelines on Accurate and Transparent Health Estimates Reporting recommendations^98^ (Supplementary Table 12) for reporting our methods and analyses. This systematic review was registered as a systematic review in the International Prospective Register of Systematic Reviews (PROSPERO, ID CRD42023421869). This study was approved by the University of Washington Institutional Review Board Committee.

### Step 1: systematic literature review and data extraction

To identify relevant studies investigating the association between long-term exposure to ambient PM_2.5_ and dementia outcomes, we initially scoured PubMed in April 2023, searching for the most recent peer-reviewed systematic review and meta-analysis studies adhering to PRISMA guidelines on long-term PM_2.5_ exposure and dementia, including both incident dementia and dementia mortality (Supplementary Information). We selected to base our systematic review Wilker et al.^13^, integrating all the publications they reviewed up to July 2022 into our literature search. Following this, we conducted comprehensive searches across multiple databases (PubMed, Embase and Web of Science) utilizing controlled vocabulary search terms associated with air pollution and dementia (detailed search strings are provided in Supplementary Table 1). This updated literature search aimed to collect newly available epidemiological evidence starting from July 2022, and the search was performed on 15 June 2023. Detailed search terms for each database can be found in the Supplementary Information. We merged the publications from the identified by Wilker et al.^13^ and the updated search, ultimately selecting 28 (refs. ^20–47^) studies for our meta-analysis based on our inclusion and exclusion criteria (see below and the Supplementary Information). The systematic review flowchart is depicted in Extended Data Fig. 1.

The systematic review was conducted independently by two reviewers who screened each study for eligibility on the basis of title and abstract. The first reviewer reviewed titles and abstracts of all identified articles. A second investigator reviewed a random sample of 10% of excluded reports for a sensitivity check. The full text of the included studies was retrieved and examined for inclusion in the meta-analysis. Finally, each full-text exclusion was verified by both reviewers. No discrepancy was found.

The searched studies were included if results were based on cohorts or pooled cohorts and reported a quantitative measurement of risk, such as HR, percentage change or *β*-coefficient associated with a range of PM_2.5_ exposure change. When multiple studies offered risk estimates from the same cohort, we incorporated only the study with the most extensive sample size or follow-up duration to avoid duplicate data inclusion. In addition, effect estimates had to include a measure of uncertainty for the effect size measure. Exposure levels had to be reported on a continuous scale of PM_2.5_ values; studies reporting estimates for categorized exposure levels were excluded. Only studies assessing the long-term impact of PM_2.5_, defined as exposure to annual means, were included. We included study cohorts with an age of 30 years and older at the beginning of the study. While our search was restricted to abstracts in English, we did include studies in all languages (unless translation was not feasible). Finally, studies were included if they assessed the impact of PM_2.5_ on incidence or mortality outcomes for any of the following types of dementia: all-cause dementia or subtypes of dementia including AD, VaD and senile dementia. We excluded animal studies. A detailed list of inclusion and exclusion criteria is included in the Supplementary Information. The source of all full-text reviewed studies and the corresponding inclusion/exclusion decisions and rationale are presented in Supplementary Table 4.

Data from eligible publications were manually extracted using a standardized template to capture the detailed information of the included studies, such as demographic characteristics, study design, location, sample size, duration of follow-up, person-years, effect size, exposure definition, PM_2.5_ exposure levels (minimum, maximum, mean, s.d., median, 25th and 75th percentiles and interquartile range), measurement methods, outcome definition, ascertainment methods, number of dementia events, risk estimates, associated uncertainties and specific confounders adjusted for in the analysis. To focus on the relationship between PM_2.5_ exposure and dementia, we extracted effect estimates exclusively from single-pollutant models, without accounting for potential effect modification or interactive effects with other air pollutants. To ensure consistency, we prioritized the most fully adjusted models reported in each study to minimize confounding. We also quantitatively assessed the risk of potential bias for each study, following the GRADE^48^ and Cochrane^76^ criteria. Supplementary Table 2 contains a full list of extracted variables.

### Step 2: estimate the risk–outcome relationships

We estimated the relationship between ambient PM_2.5_ and risk of dementia applying the MR-BRT model^16^. This model allows us to integrate RR estimates from a number of studies across different exposure ranges, and fit a nonlinear relationship between exposure and outcomes with options to impose shape constraints, such as monotonic increasing/decreasing, concave/convex and specific spline settings. In addition, the tool provides a mechanism to account for known differences in study-level characteristics by including and testing potential bias covariates related to study-level characteristics and incorporating into the final model any covariates found to introduce significant bias that would distort effect estimates (see step 3 below). The tool then additionally quantifies remaining unexplained between-study heterogeneity and estimates of uncertainty with and without consideration of this unexplained between-study heterogeneity, which forms the basis from which to calculate the BPRF, ROS and star ratings (see step 6 below). In case of data sparsity, the tool provides the ability to adjust the between-study heterogeneity parameter through a Fisher scoring correction (see step 4 below). Finally, the tool also has the ability to assess publication bias and provides a significance measure (*P* value < 0.05) (see step 5 below).

We fit curves to the data from all studies and subtype-specific studies. For all types of dementia, we included 49 extracted effect estimates from 28 studies; while for AD, we included 12 and for VaD, 6 extracted effect estimates (Extended Data Fig. 2). The number of studies for other subtypes of dementia was too small to fit individual risk–outcome curves. All exposure-response curves exhibited a harmful effect, meaning the risk of dementia increased with increasing levels of particulate matter pollution. For all curves, we placed three knots at the 25th, 50th and 75th percentile of the exposure distribution. To relax the (log-)linear assumption, our MR-BRT models were designed to flexibly adopt self-defined shape constraints (splines, monotonicity constraint and concavity/convexity constraint), allowing for a more accurate representation of the underlying relationship between PM_2.5_ and dementia. We fit the final models using a cubic spline with a linear prior on the right tail, along with monotonicity and concavity constraints. Linear-tail constraints are a common way to regularize spline behavior at the outer end of the data range. To make our results robust to knot placement, an ensemble model was created from 50 models using random knot placement by optimizing for model fit (based on a likelihood metric) and total variation (based on the highest derivative). We allowed the model to trim 10% of the data for PM_2.5_ and all-type dementia as a means to identify outliers and data points increasing between-study heterogeneity. Trimming was not implemented for the subgroup analysis due to limited data points for AD and VaD. Uncertainty estimates for the mean risk curve were generated based on a parametric bootstrapping approach. Further technical details on estimating the risk curve, spline methodology, trimming procedures, the ensemble modeling approach and uncertainty estimation are discussed elsewhere^16,18^.

We conducted stratified analyses the first of which was based on the method of dementia case diagnosis, using either active ascertainment through physician diagnosis or passive ascertainment through medical records or death certificates, as well as self-reported cases. To better understand air pollution-induced dementia risk among older adults, we conducted an additional stratified analysis focusing on cohorts with a baseline age restriction ≥60 years. Stratified analyses by subregion were also conducted to assess regional variations in the relationship between PM_2.5_ exposure and dementia. These analyses were limited to studies from North America and Europe, as insufficient data from other regions prevented model convergence.

Sensitivity analyses were further conducted to test the robustness of the model fitting. Sensitivity analysis with no-trimming and 15% trimming for all-type dementia outcomes were implemented to test the potential influence of the outliers. We further evaluated the effects of applying alternative assumptions regarding the monotonic constraint and concavity constraint (Supplementary Information).

### Step 3: assess and adjust for biases

In addition to extracting RR estimates, we extracted information regarding study design and other characteristics which could potentially bias the reported effect size. We extracted information based on common sources of bias across six domains as identified through the GRADE criteria^48^, Cochrane reviews^76^ and expert suggestions that include representativeness of the study population, exposure, outcome, reverse causation, control for confounding and selection bias. We incorporated 12 study-level covariates into our MR-BRT models, categorized under the six bias domains: (1) study population (4 factors): the representativeness of the study population (0, general population; 1, subgroup population), the method of exposure assessment (0, objective measurement; 1, self-report), restricted to older adults (0, restricted to older adults with baseline age ≥60 years; 1, broader baseline age range, strictly ≥30 but not necessarily ≥60, such as ≥40 or ≥50), geographic location (0, North America; 1, Europe; 2, Asia; 3, other regions); (2) exposure (3 factors): the method of exposure assessment (0, objective measurement; 1, self-reported exposure), the spatial resolution of exposure assessment (0, individual-level exposure estimation within a 1 × 1-km area at the individual’s residence; 1, population-level exposure estimation beyond a 1 × 1-km area at the individual’s residence) and the timing of the exposure measurement (0, time-varying exposure; 1, exposure measured only at baseline or at a fixed time point); (3) outcome (2 factors): the specific type of dementia outcome (five binary indicators for AD, VaD, non-AD dementia, senile dementia and other dementia subtype) and the method of outcome assessment (0, based on repeated cognitive testing and physician diagnosis (active ascertainment); 1, based on death certificate, medical record or self-report); (4) reverse causation (1 factor): risk of reverse causation (0, no risk of reverse causation; 1, there is a risk); (5) confounders (1 factor): risk of residual confounding (categorized into three levels: 0, low risk, controlled for age, sex, income, education and smoking; 1, medium risk, controlled for age and sex along with other covariates; and 2, high risk, controlled only for age and sex); and (6) selection bias (1 factor): the follow-up rate (classified into three levels: 0, follow-up rate exceeding 95%; 1, follow-up rate between 85% and 95%; 2, follow-up rate below 85%). See Supplementary Table 2.

We tested the risk of bias through linear regression, defining a gold standard (the preferable study design) (the Supplementary Information contains additional information on covariates extracted). Following a Lasso approach^99,100^, we ranked all covariates and added selected covariates to the model based on said ranking. Significant covariates remained in the model as the process continued. The selection process was terminated if the last added covariate was not significant. Supplementary Table 6 summarizes our assessment results of the potential bias and study quality for all included studies.

### Step 4: quantify between-study heterogeneity and adjust for within-study correlation

Differences in effect estimates across studies are referred to as between-study heterogeneity. They are commonly observed and can be introduced by methodological differences, inconsistencies across different studies, or random variations. Despite their regularity, they might lower trust in study results. After adjusting for selected bias covariates, we accounted for known heterogeneity in input study-level characteristics, but remaining unexplained heterogeneity across studies justifiably reduces trust in findings and therefore should be quantified and reported. To do so, we used a linear mixed-effects model to capture the remaining unexplained between-study heterogeneity by including a study-level random slope with variance quantified by a *γ* term. We used the Fisher information matrix^49^ to estimate the uncertainty of the heterogeneity (*γ*), which is robust to both data sparsity and the presence of within-study correlation. The uncertainty estimations not inclusive of *γ* reflect conventional estimates of uncertainty; while those that incorporating estimates of between-study heterogeneity quantified by *γ* can be used to generate the BPRF, ROS and star ratings, which are conservative estimates of the excess health risk associated with exposure to a harmful risk factor and are the primary metrics reported in this study.

### Step 5: evaluate risk of publication or reporting bias

Publication bias (the biases or selective publication of studies) is a common concern when conducting meta-analyses. In our framework, we have a twofold approach for assessing publication bias: We visually inspected funnel plots (Figs. 1–3b) displaying the residuals of the risk function and s.d. for asymmetry. In addition, we statistically tested for publication bias using Egger’s regression. Egger’s regression^50^ is a data-driven approach and is based on the detection of a correlation between the residuals and their s.e. If no publication bias was detected, the process was terminated. If we found evidence of publication of reporting bias, we reported on it but did not incorporate any correction to the risk function. This approach is standard in the general literature.

### Step 6: estimate BPRF and compute ROS

We generated the BPRF for PM_2.5_ and dementia as a combination of the mean effect as reflected by the RR curve and the estimated uncertainty including between-study heterogeneity. The BPRF is defined as the fifth quantile curve, which is the closest to the line of RR equal to 1 (the null) and can be interpreted as the smallest harmful effect at each exposure level. In other words, the BPRF represents the weakest association that is consistent with the available data after accounting for between-study heterogeneity. The larger the difference between BPRF and the null, the stronger the evidence supporting a relationship between exposure and outcome. Subsequently, we calculated the ROS, defined as the signed average log RR of the BPRF over the 15th to 85th percentiles of observed exposures^18^. A positive ROS suggests evidence of a relationship between the risk (in this study, ambient PM_2.5_) and the outcome (dementia), with higher ROS values indicating stronger evidence and more consistency in findings. Conversely, negative ROS values indicate a weaker and potentially nonsignificant risk–outcome relationship. To facilitate interpretation and comparison across studies, the BPRF framework translates ROS values into a five-star rating system: ≤0 receives one star; >0.0 to 0.14 receives two stars; >0.14 to 0.41 receives three stars; >0.41 to 0.62 receives four stars; and >0.62 receives five stars. We can interpret the star ratings as a combined measure of effect size and strength of evidence: one star denotes a weak effect and/or lack of consistent evidence across available data, with more stars signifying an increasingly stronger effect and more consistent evidence. Additionally, a zero-star rating is assigned to risk–outcome pairs lacking statistically significant association even when not including between-study heterogeneity (the conventional 95% UI without *γ* crosses the null). Risk–outcome pairs receiving a 1–5-star rating are considered candidates for inclusion in GBD.

### Statistics and reproducibility

Analyses were carried out using R v.4.0.5, Python v.3.8 and Stata v.17. To validate key aspects of the meta-regression model used in this analysis, the following packages were used, as described by Zheng et al.^18^: metafor (R package available for download at https://www.jstatsoft.org/article/view/v036i03) and dosmesreta (R package available for download at https://www.jstatsoft.org/article/view/v072c01).

This study was a secondary analysis of existing data involving systematic reviews and meta-analyses. No statistical method was used to predetermine sample size. As the study did not involve primary data collection, randomization, blinding and data exclusions are not relevant to this study and, as such, no data were excluded and we performed no randomization or blinding. We have made our data and code available to aid reproducibility.

### Reporting summary

Further information on research design is available in the Nature Portfolio Reporting Summary linked to this article.

## Supplementary information

Supplementary Information Supplementary Methods, Figs. 1–3 and Tables 1–12.

Reporting Summary

## Data Availability

The findings of this study are based on data extracted from published literature up to June 2023. We conducted systematic literature search in PubMed (https://pubmed.ncbi.nlm.nih.gov), Embase (https://www.embase.com) and Web of Science (https://www.webofscience.com), with detailed search terms provided in the Supplementary Information. We have included the full reference list for all studies incorporated in the systematic review and meta-regression. Supplementary Table 4 presents the full-text reviewed studies, along with the detailed rationale for inclusion or exclusion in the meta-regression. For studies included in the meta-regression, study characteristics and detailed extracted effect sizes are presented (Supplementary Tables 5 and 6 and Extended Data Fig. 2). The template of the data collection form is provided in Supplementary Table 2.

## Code availability

Analyses were carried out using R v.4.0.5 and Python v.3.10.9. All code used for these analyses is publicly available online (https://github.com/ihmeuw-msca/burden-of-proof/).
