# Supplementary material for: A systematic review with a Burden of Proof meta-analysis of health effects of long-term ambient fine particulate matter (PM2.5) exposure on dementia
Source: Nat Aging. 2025 Mar 21;5(5):897–908. doi: 10.1038/s43587-025-00844-y (PMC12092285; doi:10.1038/s43587-025-00844-y)
Supplement: Supplementary file 1 — Supplementary Methods, Figs. 1–3 and Tables 1–12. [file 43587_2025_844_MOESM1_ESM.pdf]

nature aging

Supplementary information https://doi.org/10.1038/s43587-025-00844-y

A systematic review with a Burden of Proof meta-analysis of health effects of long-term ambient fine particulate matter ($PM_{2.5}$) exposure on dementia

In the format provided by the authors and unedited

Table of Contents

Section 1: Data source identification and assessment 1

Section 1.1: Literature identification 1

Section 1.1.1: Identification of the systematic review and meta-regression study 1

Section 1.1.2: Identification of cohort studies via databases 2

Supplementary Table 1. Databases and search strings for the systematic review. 2

1.Section 1.2: Assessing data source eligibility 3

Supplementary Table 2. Causal criteria extraction template 5

Section 1.3: Exposure and Outcome definitions 12

Supplementary Table 3. Definitions of the exposure and the health outcome 12

Section 2: Study characteristics 14

Supplementary 4: Full-text reviewed studies (n = 59) and inclusion/exclusion decisions for meta-regression. 14

Supplementary Table 5. Study characteristic for all studies included in the systematic review and meta-regression 18

Section 3: Study quality and risk of bias assessment 21

Supplementary Table 6. Study quality for all studies (n = 28) included in the meta-regression 21

Section 4: Sensitivity analysis 23

Section 4.1: Testing sensitivity of trimming approach 23

Supplementary Fig. 1. Relationships between $PM_{2.5}$ exposure and all dementia outcomes combined from the model with (1) no trimming, and (2) 15% trimming. 23

Section 4.2: Testing sensitivity of monotonicity constraints 24

Supplementary Fig. 2. Relationships between $PM_{2.5}$ exposure and (1) all dementia outcomes combined, (2) AD, and (3) VaD as estimated from models without monotonicity constraints. 24

Section 4.3: Testing sensitivity of concavity constraints 25

Supplementary Fig. 3. Relationships between $PM_{2.5}$ exposure and (1) all dementia outcomes combined, (2) AD, and (3) VaD as estimated from models without concavity constraints. 25

Section 5: Risk curve details 26

Section 5.1: Main analysis 26

Supplementary Table 7. Risk Estimates for $PM_{2.5}$ and Dementia. 26

Section 5.2: Subgroup analysis 27

Supplementary Table 8. Risk Estimates for $PM_{2.5}$ and AD. 27

Supplementary Table 9. Risk Estimates for $PM_{2.5}$ and VaD. 29

Section 6: GATHER and PRISMA checklist 31

Supplementary Table 10. PRISMA 2020 checklist. 31

Supplementary Table 11. PRISMA 2020 abstract checklist. 35

Supplementary Table 12. GATHER checklist. 36

Section 1: Data source identification and assessment

Section 1.1: Literature identification

Our literature identification process encompassed two primary stages. Initially, we conducted literature search on PubMed to identify the most recent, peer-reviewed, and PRISMA-compliant systematic review and meta-analysis study on the association between ambient $PM_{2.5}$ and dementia, which served as the cornerstone for our systematic review. We included all the papers reviewed in this study. Subsequently, we conducted follow-up literature search on PubMed, Embase and Web of Science for epidemiological cohort studies on the relationship between ambient $PM_{2.5}$ exposure and dementia, starting from the end date of the selected systematic review study.

Section 1.1.1: Identification of the systematic review and meta-regression study

We used PubMed to identify existing meta-analysis and systematic review study for ambient $PM_{2.5}$ and dementia. The literature search is done on April 30th 2023. The PubMed search string and the criteria to identify the meta-analysis are given below.

- *PubMed search string*:

  "("Air Pollution"[Mesh] OR "Particulate Matter"[Mesh] OR "air pollution"[Title/Abstract] OR "urban air pollution"[Title/Abstract] OR "ambient air pollution"[Title/Abstract] OR "airborne particulate matter"[Title/Abstract] OR "air quality"[Title/Abstract] OR "PM2.5"[Title/Abstract])

  AND

  ("alzheimer*"[Title/Abstract] OR "dementia*"[Title/Abstract] OR "cognition"[Title/Abstract] OR "cognitive decline"[Title/Abstract] OR "Alzheimer Disease"[MeSH] OR "Dementia"[MeSH])

  AND

  ("systematic*"[Title/Abstract] OR "review"[Title/Abstract] OR "meta*"[Title/Abstract])"

- *The criteria to identify meta-analysis*:
  - PRISMA compliant – meta-analysis follows PRISMA reporting guidelines
  - Published in quality journal - journal ranks in the top two quartiles based on: https://www.scimagojr.com/journalrank.php?area=270 [scimagojr.com] (using the most appropriate subjects categories for the risk–outcome pair)
  - Incorporates inclusion criteria that are the same as or more inclusive of final inclusion criteria for risk–outcome pair
  - Most recent

We identified the study by Wilker et al. as the most recent PRISMA-compliant meta-analysis on ambient $PM_{2.5}$ and dementia, covering publications until July 2022. This systematic review yielded 38 potentially relevant citations for inclusion in our meta-analytic model. Subsequent assessment for study eligibility was conducted during the updated search step, resulting in the selection of 20 studies identified in Wilker et al. to be included in our final meta-regression analysis. More details see Extended Data Fig. 1 (PRISMA flowchart) and Section 1.1.2.

Section 1.1.2: Identification of cohort studies via databases

We conducted updated literature searches on three databases, including PubMed (https://pubmed.ncbi.nlm.nih.gov), Embase (https://www.embase.com), and Web of Science (https://www.webofscience.com), on June 25th, 2023, focusing on the period from July 1st, 2022, to June 25th, 2023. This updated search aimed to supplement information beyond the endpoint (July 2022) of the systematic review conducted in the study by Wilker et al. The search strings employed for PubMed, Embase, and Web of Science are provided below.

Supplementary Table 1. Databases and search strings for the systematic review.

Database	Search string
PubMed	"((“Air Pollution"[Mesh] OR “Particulate Matter"[Mesh] OR “air pollution"[Title/Abstract] OR “urban air pollution"[Title/Abstract] OR “ambient air pollution"[Title/Abstract] OR “airborne particulate matter"[Title/Abstract] OR “air quality"[Title/Abstract] OR “PM2.5"[Title/Abstract])<br>AND<br>("alzheimer*"[Title/Abstract] OR “dementia*"[Title/Abstract] OR “cognition” [Title/Abstract] OR “cognitive decline”[Title/Abstract] OR "Alzheimer Disease"[MeSH] OR “Dementia"[MeSH])<br>AND<br>(2022/07/01[PDAT]:2023/06/25[PDAT]) NOT (animals[MESH] NOT humans [MESH])) AND (("2022/07/01"[Date - Publication] : "2023/06/25"[Date - Publication]))”

Embase	“(‘air pollution’:ti,ab OR ‘air pollution’/exp OR ‘air pollution’ OR ‘urban air pollution’:ti,ab OR ‘ambient air pollution’:ti,ab OR ‘particulate matter’:ti,ab OR ‘particulate matter 2.5’/exp OR ‘air quality’:ti,ab OR ‘pm2.5’:ti,ab)<br>AND<br>(alzheimer*:ti,ab OR ‘dementia’:ti,ab OR 'alzheimer disease'/exp OR 'alzheimer disease' OR 'dementia' OR 'dementia'/exp OR dementia OR ‘cognition’:ti,ab OR ‘cognitive decline’:ti,ab)<br>NOT(‘animal’/exp NOT ‘human’/exp)<br>AND [01-07-2022]/sd”
Web of Science	(((TI="Air Pollution" OR AB="Air Pollution") OR ALL="Air Pollution" OR (TI=" particulate matter" OR AB="particulate matter") OR ALL="particulate matter" OR (TI="PM2.5" OR AB="PM2.5") OR (TI="air quality" OR AB="air quality"))<br>AND<br>((TI=alzheimer* OR AB=alzheimer*) OR (TI=dementia* OR AB=dementia*) OR ALL="Alzheimer Disease" OR ALL=dementia OR (TI= "cognition" OR AB= "cognition") OR (TI= "cognitive decline" OR AB= "cognitive decline"))<br>AND<br>(DOP=2022-07-01/2023-06-25))

We identified an additional 21 papers during the selection process for potential inclusion in our systematic review. This, combined with the 38 citations from Wilker et al., resulted in a total of 59 studies considered for inclusion. After thorough evaluation based on our inclusion/exclusion criteria, 28 studies were incorporated into our final meta-regression model, comprising 20 studies from Wilker et al. and 8 studies from the updated search. For a detailed overview of the systematic review process and individual study characteristics, please refer to Extended Data Fig. 1 (PRISMA flowchart), Supplementary Table 3 (Full-text reviewed studies and inclusion/exclusion decisions), and Supplementary Table 4 (Study characteristics).

1.Section 1.2: Assessing data source eligibility

See Extended Data Fig. 1 below for details on identifying, screening, and assessing eligibility for records identified through our search. The detailed inclusion and exclusion criteria used in this study is given below.

- *Inclusion criteria*:
    - Only longitudinal cohort or pooled cohort studies, with time-to-event analysis.

- Studies must report a quantitative measurement of risk, such as hazard ratio (HR), percentage changes, or beta-coefficient associated with a range of $PM_{2.5}$ exposure change. Only studies reporting an explicit exposure range will be included, while categorized exposure levels will not be.
- Include a measure of uncertainty for the effect size measure.
- Only studies on long-term exposures will be included, which is mostly evaluated by annual (rather than shorter-term) $PM_{2.5}$ exposures.
- Only studies on continuous $PM_{2.5}$ exposure will be included.
- Age 30 years and older at the beginning of the cohort
- All languages (unless we can't translate them)
- Must have incidence or mortality outcomes for any of the following: all-cause dementia, or sub-types of dementia including Alzheimer's Disease, vascular dementia, senile dementia, and other subtypes (e.g., Lewy Body disease, Frontotemporal dementia, etc.).

- *Exclusion criteria*:
  - Had occupational exposure (which does not reflect the exposure levels of the general population)
  - Were an aggregate study: meta-analysis
  - Had wrong study type: not a cohort study
  - Were duplicate study: If duplicates exist, select study to include based on exposure time, follow-up period, and covariates included in RR estimation (or other pre-determined criteria)
  - Had no measure of interest: does not report effect size (HR, percentage changes and beta-coefficient) and 95% confidence interval.
  - Had no exposure of interest: does not report any specific $PM_{2.5}$ exposure range (median/median/min/max/SD/IQR), or categorical (or binary) $PM_{2.5}$ exposure, or studies focuses on coarse particulate matter (PM_{10}) and total suspended particulate (TSP)
  - Had no outcome of interest: outcomes not related to dementia incidence/mortality.
  - Had non-general population: Studies where the population is defined by comorbidities or other traits that could interact with exposure and affect outcomes. (Our goal is to ensure that the analysis is representative of the general population to align with its use in Global Burden of Disease (GBD) studies, which will estimate the global dementia burden attributable to $PM_{2.5}$ exposure.)
  - Were animal Studies

For reports that met the inclusion criteria, data were extracted for the variables listed in Supplementary Table 1.

Supplementary Table 2. Causal criteria extraction template

**Category**	**Variable**	**Definition**
Source	seq	For RR bundle upload: A null value in the seq column indicates new data that should be inserted into the database [leave blank]
	underlying_nid	Underlying NID: Enter the underlying NID of the study (if applicable). Always talk to a data indexer if you don't know if an underlying NID is needed. They may be used for meta-analyses, certain database sources, and in some other specific cases. External Collaborators: Leave Blank
	nid	Found in GHDx, created through the epi form, or created by Data Indexer. External Collaborators: Leave Blank
	field_citation_value	IHME Zotero format or if source has NID, citation info from GHDx. External collaborators should use this column to provide a full citation of their source and include DOI
	file_path	optional; full file path of article; Only needed if source doesn't have NID, to facilitate NID creation. External Collaborators can use this field to provide a link to their source but that is optional!
R-O pair	rei	Risk: Select the risk factor, if not listed here, contact the causal criteria team. External collaborators may reach out to their assigned person of contact if they are unclear on which to choose from the list.
	acause	Outcome: Select the outcome.
Study Design	design	Study design: Specify the design of the study.
	source_type	Source type: Specify source type of study.
	study	Study Name: Enter the name of the study (e.g., Nurses' Health Study), if provided. Do not enter the title of the article.
Location	location_name	location name (from locations tab). There is a dropdown menu but it may be easier to reference to locations tab and search for your location.
	location_id	autopopulated from location_name
	smaller_site_unit	1 = if data are collected in a location more granular than the assigned location_id=1; 0 = data were not collected in a location more granular than the assigned location_id
	site_memo	open-text site field; direct copy and paste from article of all information regarding location of data collection.
Study Population	year_start	year the study was started. If article does not specify year_start and/or year_end, the general rule is to use the default of year_start = (PubYear - 3) and year_end = (PubYear - 1). Always provide (brief) documentation in note_SR if you have to make assumptions about these fields (i.e., 'Years of data

		collection not specified; used PubYear - 3; PubYear - 1.' . If information in the article makes the default seem inappropriate (e.g., the year of acceptance of the article is 2 years before the publication year), you can modify the default. Always document why you used the years you entered if you are unsure.
	year_end	year the study was finished (including most recent follow up).If article does not specify year_start and/or year_end, the general rule is to use the default of year_start = (PubYear - 3) and year_end = (PubYear - 1). Always provide (brief) documentation in note_SR if you have to make assumptions about these fields (i.e., 'Years of data collection not specified; used PubYear - 3; PubYear - 1.' . If information in the article makes the default seem inappropriate (e.g., the year of acceptance of the article is 2 years before the publication year), you can modify the default. Always document why you used the years you entered.
	age_start	ages from 1 and above must be entered as an integer. Ages <1 can be entered as decimal values, e.g., 3 days = 3/365
	age_end	ages from 1 and above must be entered as an integer. Ages <1 can be entered as decimal values, e.g., 3 days = 3/365.
	age_issue	0 = no issue flagged; 1 = issue flagged for modeler; always include explanatory notes the note SR column
	percent_male	what percent of the population is male (0-1), if pop is all female then it would be 0
	sex	Specify if the study population is exclusively male, female or both?
	sex_issue	Mark this column with a 1 to flag that there is an issue related to the sex column
	rep_geography	Were the study participants representative of the geography? 1=yes, 0=no
	rep_selection_criteria	If rep_geography is no, please specify the selection criteria of the study
	rep_prevalent_disease	Have all the study participants developed the outcomes of interest and the does the study aim to measure outcome of exposure? 1=yes 0=no (i.e., yes if for SBP-IHD paper, participants have IHD and the paper is looking at mortality due to SBP, no if for SBP-IHD paper the participants have other prevalent diseases)
Exposure	exp_def	Exposure definition: Provide a brief description of the exposure definition as reported in the study (e.g., smoking tobacco products over the past year)
	exp_timing	Timing of exposure assessment: Please specify the timing of the exposure assessment with respect to occurrence of the disease endpoint
	exp_baseline	Was the exposure assessed only at baseline?

	exp_fup_num	Specify the number of times that exposure was assessed excluding baseline
	exp_type	Which form of the exposure was included in relative risk estimation analysis?
	exp_level	Level of exposure assessment: The exposure was assessed.
	exp_method_1	Please specify the method of exposure assessment. If there are more than 1, please add in the next columns labeled "exp_method_2".
	exp_method_2	Please specify the method of exposure assessment. If there are more than 2, please add in the next columns labeled "exp_method_3".
	exp_method_3	Please specify the method of exposure assessment.
	exp_recall_type	This field describes the unit of exposure recall used in data collection. Select the correct option from the drop-down menu. If the unit is days, weeks, months, or years, please enter the number in exp_recall_type_value (next column). If the unit is 'lifetime', nothing needs to be entered in exp_recall_type_value. For example, if the study said the recall period was 4 weeks, enter 4 in exp_recall_type_value, and 'weeks' in the field exp_recall_type. If 'other' is selected, please describe in exp_recall_type_other
	exp_recall_type_value	If you entered days, weeks, months, or years in the field 'exp_recall_type', please enter the corresponding integer in this field. For example, if the study said the recall period was 4 weeks, enter 4 in exp_recall_type_value, and 'weeks' in the field exp_recall_type.
	exp_recall_type_other	If 'other' was selected in exp_recall_type, please describe the exposure recall period that the study specified (e.g., recall of exposure from 12 - 18 years).
	exp_meas_num	If the exposure level was assessed multiple times at a given time point (e.g., systolic blood pressure), specify the number of measurements at each time point.
	exp_biomarker	If the exposure level was assessed via a biomarker, specify the full name of the biomarker.
	exp_kilometer	Specify the geographical unit of measurement in kilometer (if applicable, e.g., satellite data).
	exp_instrument	Exposure assessment instrument: Specify the name of the exposure assessment instrument. For self-reported exposures, please specify the name of the questionnaire e.g., International Physical Activity Questionnaire (IPAQ).
Outcome	outcome_def	Outcome definition: Provide a brief description of the outcome as reported in the study.
	outcome_type	Outcome type: please specify if the outcome definition included incidence of or mortality from a disease endpoint

	outcome_assess_1	Method of outcome assessment: Specify the method of assessment of the study outcome. If more than 1 are appropriate, enter additional methods in the next column labeled "outcome_assess_2"
	outcome_assess_2	Method of outcome assessment: Specify the method of assessment of the study outcome. If more than 1 are appropriate, enter additional methods in the next column labeled "outcome_assess_3"
	outcome_assess_3	Method of outcome assessment: Specify the method of assessment of the study outcome.
Follow up	duration_fup_units	Units of follow up duration
	value_of_duration_fup	Enter the average length of participant follow-up.
	dropout_assess	Specify how dropout rate was defined in the study.
	dropout_rate	Dropout rate: Specify the dropout rate (%) at the end of the study. Enter on a "per 1" basis. For example: 23% is entered as “23”.
Effect Size	page_num_effect_size	Page number (where you found effect_size) from literature, or survey question where you found effect size; Use page number(s) of article, not page # of pdf
	effect_size_measure	Effect size measure: Specify the measure of effect size.
	effect_size	Effect size estimate: Provide the effect size estimate. This can also be thought of as your measure. In situations where there are 2 models (a basic and an adjusted) and the only difference are the confounders that were adjusted for, the preference will always be towards the more adjusted model. Extractors may extract the basic model as well if they deem it necessary/helpful but should indicate that this is duplicate data.
	effect_size_unit	Specify effect size unit.
	lower	Provide the lower limit of the confidence interval. Enter on a "per 1" basis. (If the CI is reported as a percent, you must convert to a decimal.) These 3 fields must all be filled in if any of them are filled in: lower, upper, uncertainty_type_value.
	upper	Provide the upper limit of the confidence interval. Enter on a "per 1" basis. (If the CI is reported as a percent, you must convert to a decimal.) These 3 fields must all be filled in if any of them are filled in: lower, upper, uncertainty_type_value.
	CI_uncertainty_type_value	This field is required if 'lower' & 'upper' are entered. This column represents the confidence level which is reported at (Eg. 95, 90, 99). These 3 fields must all be filled in if any of them are filled in: lower, upper, uncertainty_type_value.
	nonCI_uncertainty_value	Numerical value of the nonCI_uncertainty_type entered in that column. For example, if SD=5.3, you'd put 5.3 in this column, and choose SD from the drop down menu in nonCI_uncertainty_type.

	nonCI_uncertainty_type	Enter SE or SD if appropriate. For example, if SD=5.3, you'd put 5.3 in nonCI_uncertainty_value, and choose SD from the drop down menu in this column (nonCI_uncertainty_type).
	effect_size_multi_location	1 if the reported effect size is from a multi-country study and only one ffect size has been reported for all locations, otherwise 0
	pooled_cohort	1 if the reported effect size is from a pooled analysis and only pooled effect size has been reported, otherwise 0
	dose_response	Does the study support a dose-response relationship between the exposure and the outcome? External Collaborators: This field is not relevant to all study types so please judge accordingly
	dose_response_detail	If 1 was specified in the dose_response field, please specify in this field the type of evidence supporting the dose-response relationship. For example, "statistically significant p value for linear trend".
Confounders	confounders_age	if included in the relative risk estimation analysis, mark 1 for yes. Mark 0 for no
	confounders_sex	if included in the relative risk estimation analysis, mark 1 for yes. Mark 0 for no
	confounders_education	if included in the relative risk estimation analysis, mark 1 for yes. Mark 0 for no
	confounders_income	if included in the relative risk estimation analysis, mark 1 for yes. Mark 0 for no.
	confounders_smoking	if included in the relative risk estimation analysis, mark 1 for yes. Mark 0 for no.
	confounders_alcohol_use	if included in the relative risk estimation analysis, mark 1 for yes. Mark 0 for no.
	confounders_physical_activity	if included in the relative risk estimation analysis, mark 1 for yes. Mark 0 for no.
	confounders_race	if included in the relative risk estimation analysis, mark 1 for yes. Mark 0 for no.
	confounders_APOE	if included in the relative risk estimation analysis, mark 1 for yes. Mark 0 for no.
	confounders_depression	if included in the relative risk estimation analysis, mark 1 for yes. Mark 0 for no.
	confounders_coexposures	if included in the relative risk estimation analysis, mark 1 for yes. Mark 0 for no.
	confounders_dietary_components	if included in the relative risk estimation analysis, mark 1 for yes. Mark 0 for no.
	confounders_bmi	if included in the relative risk estimation analysis, mark 1 for yes. Mark 0 for no.
	confounders_hypertension	if included in the relative risk estimation analysis, mark 1 for yes. Mark 0 for no.
	confounders_diabetes	if included in the relative risk estimation analysis, mark 1 for yes. Mark 0 for no.
	confounders_hypercholesterolemia	if included in the relative risk estimation analysis, mark 1 for yes. Mark 0 for no.

	confounders_other	Use this field to list other confounders that were not mentioned in previous columns that were adjusted for in the analysis
Cohorts	cohort_person_years_exp	Please specify the person years of follow up in the exposed group
	cohort_person_years_unexp	Please specify the person years of follow up in the unexposed group
	cohort_person_years_total	Enter the total person-years of follow-up if person-years of follow up in exposed and unexposed not reported
	cohort_number_events_exp	Please specify the number of events in the exposed group
	cohort_number_events_unexp	Please specify the number of events in the unexposed group
	cohort_number_events_total	Enter the total number of events/cases if number of events in exposed and unexposed not reported
	cohort_sample_size_exp	Please specify the number of people in the exposed group if person-years of follow up in exposed not reported
	cohort_sample_size_unexp	Please specify the number of people in the unexposed group if person-years of follow up in unexposed not reported
	cohort_sample_size_total	Please specify the number of people included in the analysis if total person-years of follow up in not reported
	cohort_exposed_def	Exposed group definition: Provide a brief description of the exposed group for which the the relative risk is reported (e.g., current smokers)
	cohort_unexposed_def	Unexposed group definition: Provide a brief description of the unexposed group (i.e., the comparison group) as used in estimation of the relative risk (e.g., never smokers)
	cohort_exp_unit_rr	Exposure unit (for continuous risks): Specify the unit of exposure (e.g., grams/day).
	cohort_exp_level_rr	Exposure level in the exposed group (for continuous risks): Specify the mean/median level of exposure in the exposed group.
	cohort_unexp_level_rr	Exposure level in the unexposed group (for continuous risks): Specify the mean/median level of exposure in the unexposed group.
	cohort_exp_level_dr	Exposure level in for dose-repose RRs (for continuous risks): If the study reports dose-repose RR, please specify the level of exposure for the reported RR
	AAP_exp_conc_increment	effect size reported for how much air pollution increment (e.g. if the HR was reported for 1 $PM_{2.5}$ ug/m3 increment, then input 1)
	AAP_exp_conc_min	Minimum exposure level among the population
	AAP_exp_conc_25(Q1)	25% quantile exposure level among the population
	AAP_exp_conc_75(Q2)	75% quantile exposure level among the population

	AAP_exp_conc_max	Maximum exposure level among the population
	AAP_exp_conc_mean	Mean exposure level among the population
	AAP_exp_conc_sd	Standard deviation of the exposure level among the population
	AAP_exp_conc_median	Median exposure level among the population
	AAP_exp_conc_IQR	Interquartile range of the exposure level among the population
Covariates	cov_subpopulation	0 if generalizable--general population with reasonable exclusions, 1 analysis in subgroup such as high_risk group
	cov_exposure_population	0 for individual and 1 for population level exposure. (For air pollution risks, we consider =<1km to be individual)
	cov_exposure_selfreport	0 for measured exposure/objective measurement and 1 for self-report
	cov_exposure_study	0 for time-varying exposure and 1 for only at baseline or at a fixed time point
	cov_aged_60	0 for studies restricted to older adults with baseline age ≥ 60, 1 broader baseline age range, strictly ≥ 30 but not necessarily ≥ 60, such as ≥ 40 or ≥ 50
	cov_location	0 for North American countries (including the United States and Canada), 1 for European countries, 2 for Asian counties, and 3 for other countries
	cov_outcome_assessment	0 outcome based on physician diagnosis, 1 if outcome based on death certificate,medical record, or self-report
	cov_reverse_causation	0 no risk of reverse causation and 1 if there is a risk
	cov_confounding_uncontrolled	0 for randomization or outcome controlled for all major known confounders and age, sex, education, income, and smoking status (this varies depending on the risk). 1 for age, sex, and other critical determinants. 2 for only age and sex.
	cov_selection_bias	0 for greater than 95% follow-up, 1 for follow up of 85-95%, and 2 for less than 85% follow up. Case-control studies should be scored based on the percentages of cases and controls for which exposure data could be ascertained (avg of int/control or higher bin) *Note: this may not be the best way to capture selection bias in non RCT studies so consider creating your own true selection bias covariate if recognized as something needed
	cov_exp_measurement	Optional (use if it's helpful): multiple prospective measurements (0) vs single baseline prospective measurement (1) vs retrospective measurement/case-control (2)
	cov_AD	1 for Alzheimer's disease (AD) outcome, 0 for non-Alzheimer's disease outcome

	cov_nonAD	1 for non-Alzheimer's disease dementia (non-AD), which refers to dementia outcomes excluding AD; 0 for dementia outcomes that do not meet the criteria for non-AD dementia
	cov_VaD	1 for vascular dementia (VaD), 0 for non VaD dementia
	cov_senile	1 for senile dementia, 0 for non-senile dementia
	cov_othersubtypes	1 for other subtypes of dementia other than AD, non-AD, VaD and senile dementia; 0 for dementia outcomes that do not meet the criteria for other-subtype dementia (e.g., Lewy Body disease, Frontotemporal dementia, etc.)
Other	note_modeler	for modelers only, audience is modeler, not for correspondence
	note_sr	notes related to extraction, including assumptions, data adjustment, problems with source, any other notes that may be relevant, etc.
	extractor	uwnet id of person who extracted the data
	input_type	specify input type. Choose 1 . Use input_type tab if drop down is hard to read. This field in most ocassions should be "extracted"

Section 1.3: Exposure and Outcome definitions

In this study, the definitions for long-term ambient $PM_{2.5}$ (exposure) and dementia (outcomes) are in accordance with the health outcome definitions used in the Global Burden of Disease study

Supplementary Table 3. Definitions of the exposure and the health outcome

		Definition
Exposure	Long-term ambient $PM_{2.5}$ exposure	Exposure to ambient particulate matter pollution is defined as the population-weighted annual average mass concentration of particles with an aerodynamic diameter less than 2.5 micrometers ($PM_{2.5}$) in a cubic meter of air. This measurement is reported in $\mu g/m^3$. We only include all-source ambient $PM_{2.5}$ exposure, excluding source-specific $PM_{2.5}$. Long-term exposure is considered over a period of a year or more.
Outcome	Dementia	i. ICD codes:<br>• ICD-10 codes: F00-F03, G30, G31.1<br>F01: Vascular dementia<br>G30: Alzheimer's disease<br>G31.0: Frontotemporal dementia<br>G31.1: Senile degeneration of brain, not elsewhere classified<br>G31.83: Neurocognitive disorder with Lewy bodies<br>• ICD-9 codes: 046.1, 290.0-290.4, 294, 331.0, 331.1, 331.5, 331.82<br>290.0, 290.2, 290.3: Senile dementia<br>290.4: Vascular dementia<br>331.0: Alzheimer's disease<br>331.1: Frontotemporal dementia

		331.82: Dementia with Lewy bodies<br><br>ii. Dementia diagnosis:<br>Dementia is a progressive, degenerative, and chronic neurological disorder typified by memory impairment and other neurological dysfunctions. We use the Diagnostic and Statistical Manual of Mental Disorders III, IV or V, or ICD case definitions as the reference. The DSM-IV definition is:<br>• Multiple cognitive deficits manifested by both memory impairment and one of the following: aphasia, apraxia, agnosia, disturbance in executive functioning<br>• Must cause significant impairment in occupational functioning and represent a significant decline.<br>• Course is characterized by gradual onset and continuing cognitive decline<br>• Cognitive deficits are not due to other psychiatric conditions<br>• Deficits do not occur exclusively during the course of a delirium<br>A wide array of diagnostic and screening instruments exists, including Clinical Dementia Rating scale (CDR), Mini Mental State Examination (MMSE), and the Geriatric Mental State (GMS).<br><br>iii. Types of outcomes:<br>Both dementia incidence and mortality due to dementia are eligible in this study.<br>• Dementia incidence:<br>The number of people who develop dementia out of the total person-time at risk according to DSM or ICD criteria from a population-based survey based on evaluation by a physician or consensus meeting of physicians.<br>• Mortality due to dementia:<br>Mortality attributable to dementia, defined as unspecified dementia, Alzheimer disease, vascular dementia, and other degenerative diseases of nervous system, not elsewhere classified; according to ICD criteria from death certificates.

Section 2: Study characteristics

Supplementary 4: Full-text reviewed studies (n = 59) and inclusion/exclusion decisions for meta-regression.

First author, year	Study name/data source	Included in meta-regression	Inclusion/Exclusion rationale
Jung, 2015	Taiwan's National Insurance Research Database (NHIRD) study	Yes	Meeting all inclusion criteria
Chen, 2017	Ontario Population Health and Environment Cohort (ONPHEC) study	Yes	Distinct data source meeting all inclusion criteria
Cacciottolo, 2017[1]	Women's Health Initiative Memory Study (WHIMS) study	No	Unspecified exposure range
Carey, 2018	Clinical Practice Research Datalink (CPRD) study	Yes	Distinct data source meeting all inclusion criteria
Bishop, 2018[2]	Medicare beneficiaries	No	No measure of interest
Oudin, 2018[3]	Betula study	No	Not exposure of interest
Bowe, 2019	The US Veterans Health Administration (VA) databases cohort	Yes	Distinct data source meeting all inclusion criteria
Grande, 2019	Swedish National Study on Aging and Care in Kungsholmen (SNAC-K) study	Yes	Meeting all inclusion criteria
Cerza, 2019	Rome longitudinal study cohort	Yes	Distinct data source meeting all inclusion criteria
Lee, 2019[4]	Medicare beneficiaries in the southeastern U.S.	No	Duplicate data source with a smaller sample size compared to Shi 2021
Ilango, 2020	Ontario cohort from National Population Health Survey (NPHS) and Canadian Community Health Survey (CCHS)	Yes	Distinct data source meeting all inclusion criteria
Smargiassi, 2020	Quebec Integrated Chronic Disease Surveillance System (QICDSS) cohort	Yes	Distinct data source meeting all inclusion criteria
Ran, 2021a	Chinese Elderly Health Service (EHS) Cohort in Hong Kong	Yes	Meeting all inclusion criteria
Yuchi, 2020	Medical Services Plan (MBP) cohort	Yes	Distinct data source meeting all inclusion criteria
Klompmaker, 2020[5]	Dutch national health survey study (subset of Statistics Netherlands (CBS))	No	Duplicate data source with a smaller sample size compared to Klompmaker 2021
Shi, 2020[6]	Medicare beneficiaries	No	Duplicate data source with lower outcome assessment quality than Shi 2021
Dimakakou 2020[7]	UK Biobank	No	Duplicate data source, not study design of interest
Ho 2020[8]	The Hong Kong Census and Statistics Department's mortality dataset	No	Not study design of interest
Mortamais, 2021	The Three-City Study (3C Study)	Yes	Meeting all inclusion criteria

Shaffer, 2021	The Adult Changes in Thought (ACT) study	Yes	Distinct data source meeting all inclusion criteria
Younan, 2021	WHIMS study	Yes	Meeting all inclusion criteria (partially duplicated data source compared with Wang 2022, reporting risk estimates only for AD)
Sullivan, 2021	Monongahela-Youghiogheny Healthy Aging Team (MYHAT) cohort	Yes	Distinct data source meeting all inclusion criteria
Shi, 2021	Medicare beneficiaries	Yes	Meeting all inclusion criteria
Klompmaker, 2021	CBS cohort	Yes	Meeting all inclusion criteria
Nunez, 2021[9]	New York Department of Health Statewide Planning and Research Cooperative System	No	Not study design of interest
Rhew, 2021[10]	The State Center for Health Statistics	No	Not exposure of interest
Ran, 2021b[11]	Chinese EHS cohort in Hong Kong	No	Duplicate data source, unspecified exposure range
Van Wijngaarden, 2021[12]	New York State Statewide Planning and Research Cooperative System (SPARCS)	No	Not exposure of interest
Astrom, 2021[13]	Betula cohort	No	Unspecified exposure range
Semmens, 2022	Ginkgo Evaluation of Memory Study (GEMS)	Yes	Meeting all inclusion criteria
Wang, 2022	WHIMS Epidemiology of Cognitive Health Outcomes (WHIMS-ECHO) study	Yes	Meeting all inclusion criteria (partially duplicated data source compared with Younan 2021, reporting risk estimates for all-cause dementia)
So, 2022	Danish nationwide administrative cohort	Yes	Distinct data source meeting all inclusion criteria
Chen, 2022a	UK biobank	Yes	Meeting all inclusion criteria
Yang, 2022	Zhejiang cohort in China	Yes	Distinct data source meeting all inclusion criteria
Wood, 2022	English Longitudinal Study of Ageing (ELSA) cohort	Yes	Distinct data source meeting all inclusion criteria
Trevenen, 2022	The Health in Men Study (HIMS)	Yes	Distinct data source meeting all inclusion criteria
Andersen, 2022	"Effects of Low-Level Air Pollution: A Study in Europe" (ELAPSE) pooled cohort	Yes	Distinct data source meeting all inclusion criteria
Chen, 2022b[14]	WHIMS study	No	Duplicate data source, unspecified exposure range
Letellier, 2022[15]	3C Study	No	Duplicate data source, reporting risk estimates for reduced $PM_{2.5}$ exposure
He, 2022[16]	Zhejiang Major Public Health Surveillance (ZJMPHS) Program	No	Not exposure of interest (categorical exposure)
Parra, 2022[17]	UK Biobank	No	Duplicate data source with a smaller sample size compared to Chen 2022
Ma, 2022[18]	UK Biobank	No	Duplicate data source with a smaller sample size compared to Chen 2022
Raichlan, 2022[19]	UK Biobank	No	Duplicate data source with a smaller sample size compared to Chen 2022

Xie, 2022[20]	China Family Panel Studies (CFPS)	No	Not study design of interest
Mukadam, 2022[21]	UK Biobank	No	Duplicate data source, not exposure of interest
Yan, 2022[22]	NHIRD study	No	Duplicate data source, not exposure of interest
Simos, 2022[23]	Belgium cohort	No	No measure of interest
Wu, 2022[24]	SNAC-K study	No	Duplicate data source, not population of interest
De Crom, 2023	Rotterdam cohort	Yes	Distinct data source meeting all inclusion criteria
Zhu, 2023	Ningbo cohort in China	Yes	Distinct data source meeting all inclusion criteria
Yu, 2023	Sacramento Area Latino Study on Aging (SALSA)	Yes	Distinct data source meeting all inclusion criteria
Andersson, 2023	Betula study	Yes	Meeting all inclusion criteria
Zhang, 2023[25]	UK biobank	No	Duplicate data source with a smaller sample size compared to Chen 2022
Franz, 2023[26]	Vietnam Era Twin Study of Aging	No	Not outcome of interest
Cole-Hunter, 2023[27]	ELAPSE pooled cohort	No	Duplicate data sources, not outcome of interest
Ilango, 2023[28]	GEMS cohort	No	Duplicate data source, focused on joint effects of social environment and air pollution
Shi, 2023[29]	Medicare beneficiaries	No	Duplicate data source, focused on $PM_{2.5}$ components, with a shorter follow-up period and shorter exposure windows
Hu, 2023[30]	UK biobank	No	Duplicate data source, not study design of interest
Mork, 2023[31]M	Medicare beneficiaries	No	No measure of interest

Notes: A total of 59 studies were included in the full-text review, of which 28 studies were incorporated into the meta-regression. The remaining studies were excluded for the following reasons: no exposure of interest (n = 6), unspecified exposure range (n = 4), no study design of interest (n = 5), non-target population (n = 1), no outcome of interest (n = 2), and no measure of interest (n = 3). Additionally, 10 studies were excluded due to duplicate data sources. For a detailed overview of the study selection process, refer to the PRISMA flowchart (Extended Data Fig. 1). Full citations for included studies are provided in the main text reference list, while the complete bibliography of excluded studies is listed below.

1. Cacciottolo, M. *et al.* Particulate air pollutants, APOE alleles and their contributions to cognitive impairment in older women and to amyloidogenesis in experimental models. *Transl. Psychiatry* **7**, e1022–e1022 (2017).
2. Bishop, K., Ketcham, J. & Kuminoff, N. *Hazed and Confused: The Effect of Air Pollution on Dementia*. w24970 http://www.nber.org/papers/w24970.pdf (2018) doi:10.3386/w24970.
3. Oudin, A., Segersson, D., Adolfsson, R. & Forsberg, B. Association between air pollution from residential wood burning and dementia incidence in a longitudinal study in Northern Sweden. *PLOS ONE* **13**, e0198283 (2018).
4. Lee, M., Schwartz, J., Wang, Y., Dominici, F. & Zanobetti, A. Long-term effect of fine particulate matter on hospitalization with dementia. *Environ. Pollut.* **254**, 112926 (2019).
5. Klompmaker, J. O. *et al.* Surrounding green, air pollution, traffic noise exposure and non-accidental and cause-specific mortality. *Environ. Int.* **134**, 105341 (2020).
6. Shi, L. *et al.* Long-term effects of PM2·5 on neurological disorders in the American Medicare population: a longitudinal cohort study. *Lancet Planet. Health* **4**, e557–e565 (2020).
7. Dimakakou, E., Johnston, H. J., Streftaris, G. & Cherrie, J. W. Is Environmental and Occupational Particulate Air Pollution Exposure Related to Type-2 Diabetes and Dementia? A Cross-Sectional Analysis of the UK Biobank. *Int. J. Environ. Res. Public. Health* **17**, 9581 (2020).

8. Ho, H. C., Fong, K. N. K., Chan, T.-C. & Shi, Y. The associations between social, built and geophysical environment and age-specific dementia mortality among older adults in a high-density Asian city. *Int. J. Health Geogr.* **19**, 53 (2020).
9. Nunez, Y. *et al.* Fine Particle Exposure and Clinical Aggravation in Neurodegenerative Diseases in New York State. *Environ. Health Perspect.* **129**, 027003 (2021).
10. Rhew, S. H., Kravchenko, J. & Lyerly, H. K. Exposure to low-dose ambient fine particulate matter PM2.5 and Alzheimer's disease, non-Alzheimer's dementia, and Parkinson's disease in North Carolina. *PLOS ONE* **16**, e0253253 (2021).
11. Ran, J. *et al.* The joint association of physical activity and fine particulate matter exposure with incident dementia in elderly Hong Kong residents. *Environ. Int.* **156**, 106645 (2021).
12. Van Wijngaarden, E. *et al.* Neurodegenerative hospital admissions and long-term exposure to ambient fine particle air pollution. *Ann. Epidemiol.* **54**, 79-86.e4 (2021).
13. Åström, D. O., Adolfsson, R., Segersson, D., Forsberg, B. & Oudin, A. Local Contrasts in Concentration of Ambient Particulate Air Pollution (PM2.5) and Incidence of Alzheimer's Disease and Dementia: Results from the Betula Cohort in Northern Sweden. *J. Alzheimers Dis.* **81**, 83–85 (2021).
14. Chen, C. *et al.* B vitamin intakes modify the association between particulate air pollutants and incidence of all-cause dementia: Findings from the Women's Health Initiative Memory Study. *Alzheimers Dement.* **18**, 2188–2198 (2022).
15. Letellier, N. *et al.* Air quality improvement and incident dementia: Effects of observed and hypothetical reductions in air pollutant using parametric g-computation. *Alzheimers Dement.* **18**, 2509–2517 (2022).
16. He, F. *et al.* Impact of air pollution exposure on the risk of Alzheimer's disease in China: A community-based cohort study. *Environ. Res.* **205**, 112318 (2022).
17. Parra, K. L., Alexander, G. E., Raichlen, D. A., Klimentidis, Y. C. & Furlong, M. A. Exposure to air pollution and risk of incident dementia in the UK Biobank. *Environ. Res.* **209**, 112895 (2022).
18. Ma, H. *et al.* Long-term exposure to low-level air pollution, genetic susceptibility and risk of dementia. *Int. J. Epidemiol.* **52**, 738–748 (2023).
19. Raichlen, D. A. *et al.* Association of Physical Activity with Incidence of Dementia Is Attenuated by Air Pollution. *Med. Sci. Sports Exerc.* **54**, 1131–1138 (2022).
20. Xie, J. & Lu, C. Is there a casual relation between air pollution and dementia? *Environ. Sci. Pollut. Res. Int.* **30**, 23248–23262 (2023).
21. Mukadam, N., Marston, L., Lewis, G. & Livingston, G. Risk factors, ethnicity and dementia: A UK Biobank prospective cohort study of White, South Asian and Black participants. *PLoS ONE* **17**, (2022).
22. Yan, Y.-H. *et al.* Long-term exposure to particulate matter was associated with increased dementia risk using both traditional approaches and novel machine learning methods. *Sci. Rep.* **12**, 17130 (2022).
23. Simos, J. Long-term exposure to residential greenness and neurodegenerative disease mortality among older adults: a 13-year follow-up cohort study. *Environ. Risques Sante* **21**, 382–385 (2022).
24. Wu, J. *et al.* Air pollution as a risk factor for Cognitive Impairment no Dementia (CIND) and its progression to dementia: A longitudinal study. *Environ. Int.* **160**, 107067 (2022).
25. Zhang, Z. *et al.* Associations of Air Pollution and Genetic Risk With Incident Dementia: A Prospective Cohort Study. *Am. J. Epidemiol.* **192**, 182–194 (2023).
26. Franz, C. E. *et al.* Associations Between Ambient Air Pollution and Cognitive Abilities from Midlife to Early Old Age: Modification by APOE Genotype. *J. Alzheimers Dis. JAD* **93**, 193–209 (2023).
27. Cole-Hunter, T. *et al.* Long-term air pollution exposure and Parkinson's disease mortality in a large pooled European cohort: An ELAPSE study. *Environ. Int.* **171**, (2023).
28. Ilango, S. D. *et al.* An Examination of the Joint Effect of the Social Environment and Air Pollution on Dementia among US Older Adults. *Environ. Epidemiol.* **7**, E250 (2023).
29. Shi, L. *et al.* Incident dementia and long-term exposure to constituents of fine particle air pollution: A national cohort study in the United States. *Proc. Natl. Acad. Sci. U. S. A.* **120**, (2023).
30. Hu, H.-Y. *et al.* Residential greenness and risk of incident dementia: A prospective study of 375,342 participants. *Environ. Res.* **216**, (2023).
31. Mork, D., Braun, D. & Zanobetti, A. Time-lagged relationships between a decade of air pollution exposure and first hospitalization with Alzheimer's disease and related dementias. *Environ. Int.* **171**, (2023).

Supplementary Table 5. Study characteristic for all studies included in the systematic review and meta-regression

S/N	First author, year	Study name	Region	Population Size (n)	Baseline age (y)	Follow-up period (y)	Percentage male (%)	$PM_{2.5}$ mean /median* ($\mu g/m^3$)	Exposure averaging period	Outcome endpoint	Dementia case identification	All-cause dementia (n)	AD (n)	VaD (n)
1	Jung, 2015	NHIRD study	Taiwan, China	95,609	≥ 65	10	0.54	33.6	Baseline annual average	Incidence	Medical records	--	1,399	--
2	Chen, 2017	ONPHEC study	Ontario, Canada	2,006,639	≥ 55	13	0.47	10.4	Time-varying 5-year average	Incidence	Medical records	257,816	--	--
3	Carey, 2018	CPRD study	London, UK	130,978	≥ 50	9	0.50	15.7	Baseline annual average	Incidence	Medical records	2,181	848	634
4	Bowe, 2019	US VA study	Umea, Sweden	4,522,160	≥ 40	11	0.94	11.9*	Baseline annual average	Mortality	Death certificates	103,268	--	--
5	Grande, 2019	SNAC-K study	USA	2,927	≥ 60	13	0.37	8.5	Time-varying 5-year average	Incidence &Mortality	Physician diagnosis	364	218[†]	70
6	Cerza, 2019	Rome cohort	Rome, Italy	350,844	≥ 65	13	0.42	19.7	Fixed annual average (2010)	Incidence	Medical records	21,548	7,671	7,500
7	Ilango, 2020	NPHS & CCHS study	Ontario, Canada	34,391	≥ 45	18	0.42	8.6	Time-varying 3-year average	Incidence	Medical records	2,559	--	--
8	Smargiassi, 2020	QICDSS study	Quebec, Canada	1,807,133	≥ 65	13	0.45	7.6	Time-varying 2-year average	Incidence	Medical records	199,826	--	--
9	Ran 2021	HK EHS study	HK, China	59,349	≥ 65	14	0.34	35.2	Baseline 3-year average	Incidence	Medical records	1,183	655	334

10	Yuchi, 2020	MBP study	USA	633,949	≥ 45	10	0.47	4.1*	Time-varying annual average	Incidence	Medical records	--	--	--
11	Mortamais, 2021	3C study	Dijon; Bordeaux; Montpellier, France	7,066	≥ 65	14	0.38	21.9	Time-varying 10-year average	Incidence	Physician diagnosis	791	541	--
12	Shaffer, 2021	ACT study	WA, USA	4,166	≥ 65	25	0.42	10.1	Time-varying 10-year average	Incidence	Physician diagnosis	1,136	921	--
13	Younan, 2021	WHIMS study	USA	5,798	≥ 65	15	0.00	12.7	Time-varying annual average	Incidence	Physician diagnosis	--	130	--
14	Sullivan, 2021	MYHAT study	PA, USA	1,572	≥ 65	9	0.38	1.5	Time-varying 1-/5-year average	Incidence	CDR※ performed by trained interviewers	108	--	--
15	Shi, 2021	Medicare study	USA	12,233,371	≥ 65	19	0.41	9.3	Time-varying 5-year average	Incidence	Medical records	2,025,103	--	--
16	Klompmaker, 2021	CBS study	Netherlands	10,481,566	≥ 30	6	0.49	16.8*	Fixed annual average	Mortality	Death certificates	57,122	--	--
17	Semmens, 2022	GEMS study	NC; MD; CA; PA, USA	2,564	≥ 75	9	0.54	18.4	Baseline 5-/10-/20-year average	Incidence	Physician diagnosis	324	--	--
18	Wang, 2022	WHIMS-ECHO study	USA	2,239	≥ 74	11	0.00	13.3	Baseline 3-year average	Incidence	Physician diagnosis	398	--	--
19	So, 2022	Danish national cohort	Denmark	3,083,227	≥ 30	18	0.48	12.4	Baseline annual average	Mortality	Death certificates	41,141	--	--

20	De Crom, 2023	Rotterdam cohort	Rotterdam, Netherlands	7,551	≥ 49	9	0.41	16.8	Baseline annual average	Incidence	Physician diagnosis	545	--	--
21	Chen, 2022	UK biobank cohort	UK	459,844	≥ 40	15	--	10.1*	Fixed annual average (2010)	Incidence &Mortality	Medical records & Death certificates	5,950	2,535	1,493
22	Yang, 2022	Zhejiang cohort	Zhejiang, China	1,545	≥ 60	3	0.48	36.2	Baseline 5-year average	Incidence	Physician diagnosis	--	--	--
23	Wood, 2022	ELSA cohort	UK	8,525	≥ 50	14	0.45	12.1	Baseline annual average	Incidence	Self-reported	389	--	--
24	Trevenen, 2022	HIMS cohort	Perth, Australia	11,243	≥ 65	24	1.00	4.5	Baseline and time-varying annual average	Incidence	Medical records & Death certificates	3,053	1,670	355
25	Zhu, 2023	Ningbo cohort	Ningbo, China	29,025	≥ 40	8	0.41	34.5*	Baseline annual average	Incidence	Medical records & Death certificates	--	128	--
26	Yu, 2023	SALSA cohort	CA, USA	1,612	≥ 60	10	0.42	12.8	Baseline 5-year average and time-varying 1-/3-/5-year average	Incidence	Physician diagnosis	104	--	--
27	Andersson, 2023	Betula study	Umea, Sweden	1,846	≥ 55	21	0.44	6.8	Baseline annual average	Incidence	Medical records	348	--	--
28	Andersen, 2022	ELAPSE pooled cohort	Western Europe	271,720	--	19.7 (mean)	0.31	16.2	Fixed annual average	Mortality	Death certificates	900	--	--

Notes: ※: CDR refers to Clinical Dementia Rating. †: Effect estimates for AD were not reported in Grande 2019. If a study reported multiple risk estimates for the same dementia outcome across different exposure windows, only the estimate from the longest exposure window was selected for the meta-analysis (see Extended Data Fig. 2 for detailed extracted risk estimates).

Section 3: Study quality and risk of bias assessment

Each study meeting the inclusion criteria was reviewed by the first reviewer to evaluate bias indicators listed in the extraction template (Supplementary Table 2), while a second reviewer cross-verified the accuracy and completeness of all extracted data. These bias indicators were then categorized as binary or categorical covariates and considered in risk curve estimations (Supplementary Table 6). Upon identifying significant indicators, only these relevant covariates were retained in the final MR-BRT models.

Supplementary Table 6. Study quality for all studies (n = 28) included in the meta-regression

S/N	First author, year	Study name	cov_subpopulation	cov_exposure_population	cov_aged_60	cov_location	cov_exposure_selfreport	cov_exposure_study	cov_outcome_assessment	cov_confounding_uncontrolled	cov_selection_bias
1	Jung, 2015	NHIRD study	0	0	0	2	0	1	1	1	0
2	Chen, 2017	ONPHEC study	0	0	1	0	0	0	1	1	2
3	Carey, 2018	CPRD study	0	0	1	1	0	1	1	0	1
4	Bowe, 2019	US VA study	1	1	1	0	0	1	1	0	0
5	Grande, 2019	SNAC-K study	0	0	0	0	0	0	0	0	2
6	Cerza, 2019	Rome cohort	0	0	0	1	0	1	1	1	0
7	Ilango, 2020	NPHS & CCHS study	0	0	1	0	0	1	1	0	2
8	Smargiassi, 2020	QICDSS study	0	0	0	0	0	0	1	1	2
9	Ran 2021	HK EHS study	0	0	0	2	0	0	1	0	1
10	Yuchi, 2020	MBP study	0	0	1	0	0	1	1	0	2
11	Mortamais, 2021	3C study	0	0	0	1	0	0	0	0	2
12	Shaffer, 2021	ACT study	0	0	0	0	0	0	0	0	0
13	Younan, 2021	WHIMS study	1	0	0	0	0	0	0	0	1
14	Sullivan, 2021	MYHAT study	0	1	0	0	0	0	0	0	1
15	Shi, 2021	Medicare study	0	0	0	0	0	1	1	1	2
16	Klompmaker, 2021	CBS study	0	1	1	1	0	1	1	1	0
17	Semmens, 2022	GEMS study	0	1	0	0	0	1	0	0	0
18	Wang, 2022	WHIMS-ECHO study	0	1	0	0	0	1	0	0	1
19	So, 2022	Danish national cohort	0	0	1	1	0	1	1	0	1
20	De Crom, 2023	Rotterdam cohort	0	0	1	1	0	1	0	1	0
21	Chen, 2022	UK biobank cohort	0	0	1	1	0	1	1	1	0
22	Yang, 2022	Zhejiang cohort	0	0	0	2	0	1	0	0	1
23	Wood, 2022	ELSA cohort	0	0	1	1	0	1	1	0	2

24	Trevenen, 2022	HIMS cohort	0	0	0	3	0	0	1	1	0
25	Zhu, 2023	Ningbo cohort	0	0	1	2	0	1	1	0	2
26	Yu, 2023	Sacramento Latino cohort	1	0	0	0	0	0	0	0	0
27	Andersson, 2023	Betula study	0	0	1	1	0	1	1	1	1
28	Andersen, 2022	ELAPSE pooled cohort	0	0	1	1	0	1	1	1	2

Notes: For all studies, exposure assessments were derived from air pollution models (cov_exposure_selfreport = 0), and there was no risk of reverse causation in the longitudinal studies, as the exposure window preceded the outcome diagnosis (cov_reserve_causation = 0).

Section 4: Sensitivity analysis

Section 4.1: Testing sensitivity of trimming approach

We conducted sensitivity analysis for the trimming approach to evaluate the impact of our 10% trimming on the shape of the risk curves for $PM_{2.5}$ and all dementia outcomes combined. In our final model, 10% of the input data was trimmed to eliminate extreme values and minimize publication bias. We executed two preliminary models: one without any trimming and another with 15% trimming. All other model specifications remained consistent with our final model. Below are the outcomes from both models: the model without trimming and the model with 15% trimming (Supplementary Fig. 1).

(1) No trimming

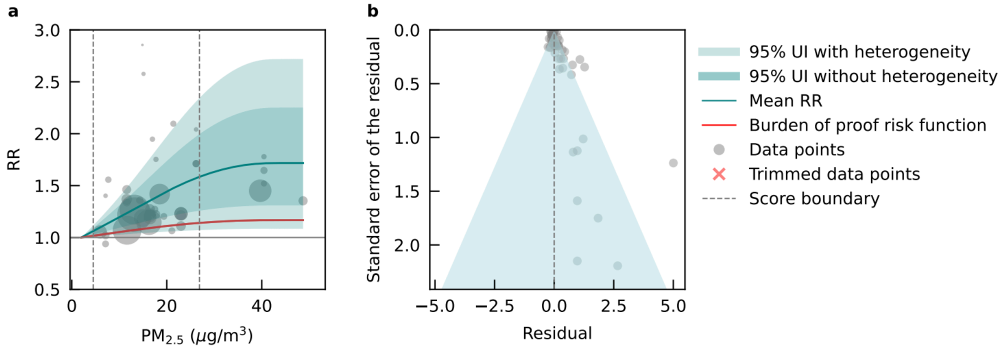

(2) 15% trimming

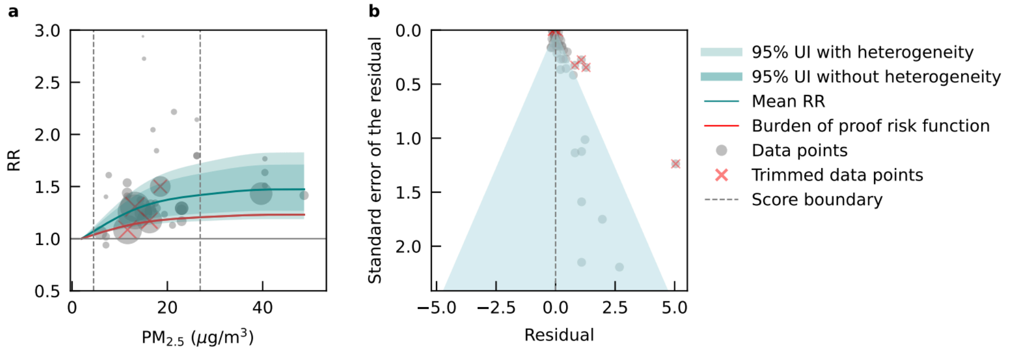

Supplementary Fig. 1. Relationships between $PM_{2.5}$ exposure and all dementia outcomes combined from the model with (1) no trimming, and (2) 15% trimming. All other model specifications are consistent with the final models. **a**, RR function. The solid green line represents the mean RR at each exposure level, and the solid red line represents the Burden of proof risk function. The dark green shaded area indicates the 95% uncertainty interval (UI) without accounting for between-study heterogeneity, while the light green shaded area represents the 95% UI accounting for between-study heterogeneity. The size of the data points is proportional to the inverse of the standard deviation of the effect estimates, where larger points indicate higher precision in the effect estimates. **b**, A modified funnel plot showing the residuals (relative to zero) on the x axis and the estimated s.d. (inclusive of between-study heterogeneity) on the y axis.

Section 4.2: Testing sensitivity of monotonicity constraints

We examined the impact of the monotonicity constraint on the shape of the risk curves for $PM_{2.5}$ and dementia to evaluate the sensitivity of our final model to this modeling assumption. This constraint ensured that the mean risk curve was non-decreasing where the relationship is expected to be harmful. Preliminary model was run with the same inputs and model specifications of the final model but without the monotonicity constraint, and the result was compared to those of the final models. The result can be found below (Supplementary Fig. 2).

(1) All dementia outcomes combined:

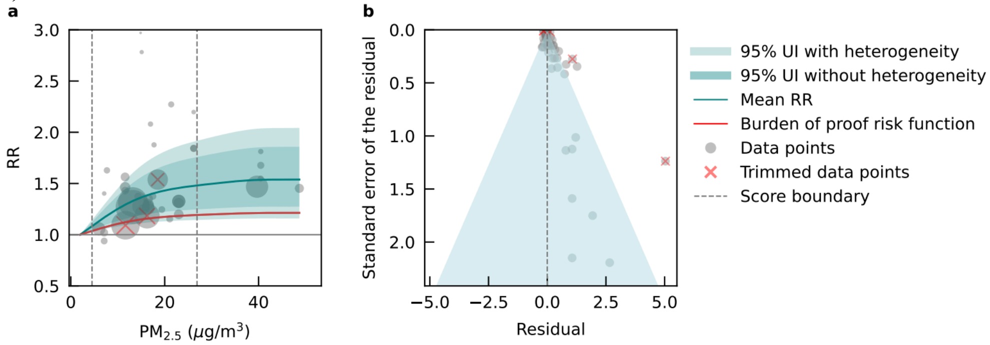

(2) AD:

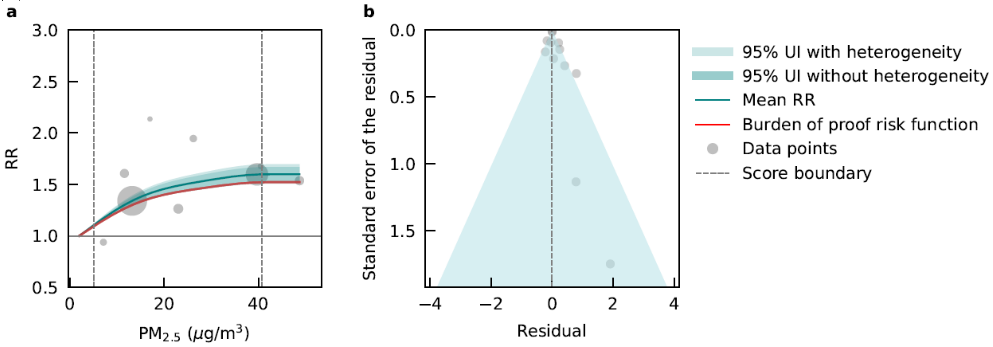

(3) VaD:

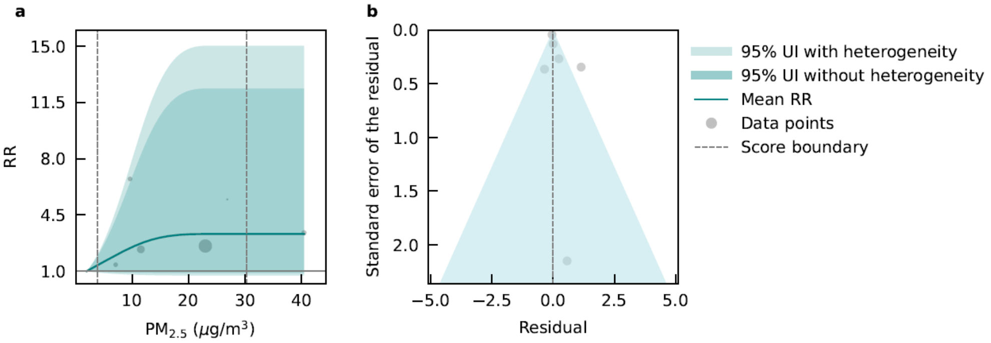

Supplementary Fig. 2. Relationships between $PM_{2.5}$ exposure and (1) all dementia outcomes combined, (2) AD, and (3) VaD as estimated from models without monotonicity constraints. All other model specifications are consistent with the final models. **a**, RR function. The solid green line represents the mean RR at each exposure level, and the solid red line represents the Burden of proof risk function. The dark green shaded area indicates the 95% uncertainty interval (UI) without accounting for between-study heterogeneity, while the light green shaded area represents the 95% UI accounting for between-study heterogeneity. The size of the data points is proportional to the inverse of the standard deviation of the effect estimates, where larger points indicate higher precision in the effect estimates. **b**, A modified funnel plot showing the residuals (relative to zero) on the x axis and the estimated s.d. that includes reported s.d. and between-study heterogeneity on the y axis. No trimming was performed for AD and VaD due to the limited dataset available.

Section 4.3: Testing sensitivity of concavity constraints

We examined the impact of the concavity constraint on the shape of the risk curves for $PM_{2.5}$ and dementia to evaluate the sensitivity of our final model to this modeling assumption. Preliminary model was run with the same inputs and model specifications of the final model but without the concavity constraint, and the result was compared to those of the final models. The result can be found below (Supplementary Fig. 3).

(1) All dementia outcomes combined

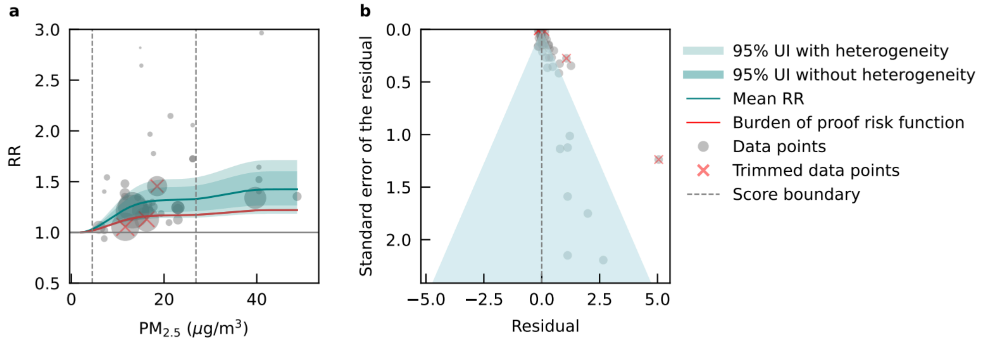

(2) AD

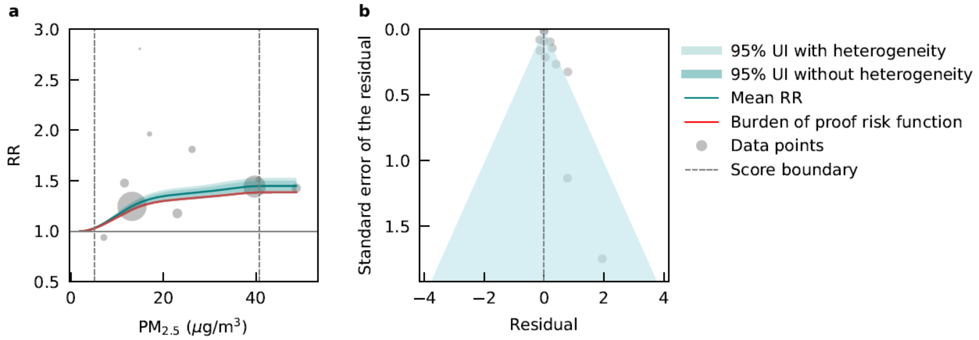

(3) VaD

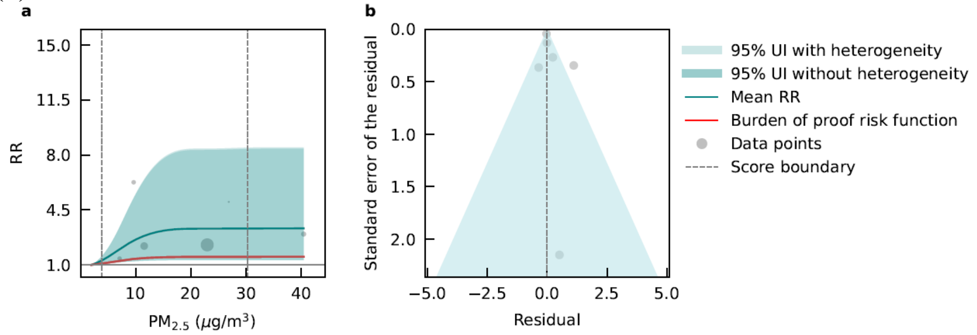

Supplementary Fig. 3. Relationships between $PM_{2.5}$ exposure and (1) all dementia outcomes combined, (2) AD, and (3) VaD as estimated from models without concavity constraints. All other model specifications are consistent with the final models. **a**, RR function. The solid green line represents the mean RR at each exposure level, and the solid red line represents the Burden of proof risk function. The dark green shaded area indicates the 95% uncertainty interval (UI) without accounting for between-study heterogeneity, while the light green shaded area represents the 95% UI accounting for between-study heterogeneity. The size of the data points is proportional to the inverse of the standard deviation of the effect estimates, where larger points indicate higher precision in the effect estimates. **b**, A modified funnel plot showing the residuals (relative to zero) on the x axis and the estimated s.d. (inclusive of between-study heterogeneity) on the y axis. No trimming was performed for AD and VaD due to the limited dataset available.

Section 5: Risk curve details

Section 5.1: Main analysis

Supplementary Table 7. Risk Estimates for $PM_{2.5}$ and Dementia. Mean relative risk measures of dementia risk across ambient $PM_{2.5}$ exposure Relative risk (RR) based on the mean relative risk function (along with the 95% uncertainty interval (UI) when not incorporating between-study heterogeneity and when accounting for between-study heterogeneity), presented at every 1 μg/m^3 of $PM_{2.5}$ from 2 to 48 μg/m^3. The mean RRs are calculated in comparison to a reference $PM_{2.5}$ level of 2.0 μg/m^3.

Ambient $PM_{2.5}$ level (μg/m^3)	RR (95% UI without between-study heterogeneity)	RR (95% UI with between-study heterogeneity)
2.0	1 (ref.)	1 (ref.)
3.0	1.03 (1.02, 1.05)	1.03 (1.01, 1.06)
4.0	1.07 (1.04, 1.10)	1.07 (1.03, 1.11)
5.0	1.10 (1.06, 1.15)	1.10 (1.04, 1.17)
6.0	1.13 (1.07, 1.19)	1.13 (1.05, 1.23)
7.0	1.16 (1.09, 1.24)	1.16 (1.06, 1.29)
8.0	1.19 (1.11, 1.29)	1.19 (1.07, 1.35)
9.0	1.22 (1.12, 1.33)	1.22 (1.08, 1.40)
10.0	1.25 (1.14, 1.38)	1.25 (1.09, 1.45)
11.0	1.28 (1.15, 1.42)	1.28 (1.10, 1.51)
12.0	1.30 (1.16, 1.46)	1.30 (1.11, 1.55)
13.0	1.33 (1.17, 1.49)	1.33 (1.12, 1.60)
14.0	1.35 (1.18, 1.53)	1.35 (1.12, 1.64)
15.0	1.36 (1.19, 1.56)	1.36 (1.13, 1.68)
16.0	1.38 (1.20, 1.59)	1.38 (1.13, 1.71)
17.0	1.40 (1.21, 1.61)	1.40 (1.14, 1.74)
18.0	1.41 (1.22, 1.63)	1.41 (1.14, 1.77)
19.0	1.42 (1.22, 1.65)	1.42 (1.15, 1.80)
20.0	1.44 (1.23, 1.67)	1.43 (1.15, 1.82)
21.0	1.45 (1.23, 1.68)	1.44 (1.15, 1.84)
22.0	1.46 (1.24, 1.70)	1.45 (1.15, 1.86)
23.0	1.46 (1.24, 1.71)	1.46 (1.16, 1.87)
24.0	1.47 (1.24, 1.72)	1.46 (1.16, 1.89)
25.0	1.47 (1.25, 1.73)	1.47 (1.16, 1.90)

26.0	1.48 (1.25, 1.74)	1.48 (1.16, 1.91)
27.0	1.48 (1.25, 1.75)	1.48 (1.16, 1.92)
28.0	1.49 (1.25, 1.76)	1.49 (1.17, 1.94)
29.0	1.49 (1.26, 1.77)	1.49 (1.17, 1.95)
30.0	1.50 (1.26, 1.78)	1.50 (1.17, 1.96)
31.0	1.50 (1.26, 1.79)	1.50 (1.17, 1.97)
32.0	1.51 (1.26, 1.80)	1.51 (1.17, 1.98)
33.0	1.51 (1.27, 1.80)	1.51 (1.17, 1.99)
34.0	1.52 (1.27, 1.81)	1.52 (1.18, 2.00)
35.0	1.52 (1.27, 1.82)	1.52 (1.18, 2.01)
36.0	1.52 (1.27, 1.83)	1.52 (1.18, 2.02)
37.0	1.53 (1.27, 1.83)	1.53 (1.18, 2.03)
38.0	1.53 (1.28, 1.84)	1.53 (1.18, 2.03)
39.0	1.53 (1.28, 1.84)	1.53 (1.18, 2.04)
40.0	1.54 (1.28, 1.84)	1.54 (1.18, 2.04)
41.0	1.54 (1.28, 1.85)	1.54 (1.18, 2.05)
42.0	1.54 (1.28, 1.85)	1.54 (1.18, 2.05)
43.0	1.54 (1.28, 1.85)	1.54 (1.18, 2.05)
44.0	1.54 (1.28, 1.85)	1.54 (1.18, 2.05)
45.0	1.54 (1.28, 1.85)	1.54 (1.18, 2.05)
46.0	1.54 (1.28, 1.85)	1.54 (1.18, 2.05)
47.0	1.54 (1.28, 1.85)	1.54 (1.18, 2.05)
48.0	1.54 (1.28, 1.85)	1.54 (1.18, 2.05)

Section 5.2: Subgroup analysis

Supplementary Table 8. Risk Estimates for $PM_{2.5}$ and AD. Mean relative risk measures of AD risk across ambient $PM_{2.5}$ exposure Relative risk (RR) based on the mean relative risk function (along with the 95% uncertainty interval (UI) when not incorporating between-study heterogeneity and when accounting for between-study heterogeneity), presented at every 1 μg/m^3 of $PM_{2.5}$ from 2 to 48 μg/m^3. The mean RRs are calculated in comparison to a reference $PM_{2.5}$ level of 2.0 ug/m^3.

Ambient $PM_{2.5}$ level (μg/m^3)	RR (95% UI without between-study heterogeneity)	RR (95% UI with between-study heterogeneity)
2.0	1 (ref.)	1 (ref.)

3.0	1.04 (1.03, 1.04)	1.04 (1.03, 1.04)
4.0	1.07 (1.06, 1.08)	1.07 (1.06, 1.08)
5.0	1.10 (1.09, 1.11)	1.10 (1.09, 1.12)
6.0	1.14 (1.12, 1.15)	1.14 (1.12, 1.15)
7.0	1.17 (1.15, 1.18)	1.17 (1.15, 1.19)
8.0	1.20 (1.18, 1.22)	1.20 (1.17, 1.23)
9.0	1.23 (1.21, 1.25)	1.23 (1.20, 1.26)
10.0	1.26 (1.23, 1.28)	1.26 (1.22, 1.29)
11.0	1.29 (1.26, 1.31)	1.29 (1.25, 1.32)
12.0	1.31 (1.28, 1.34)	1.31 (1.27, 1.35)
13.0	1.34 (1.30, 1.37)	1.34 (1.29, 1.38)
14.0	1.36 (1.32, 1.40)	1.36 (1.31, 1.41)
15.0	1.38 (1.34, 1.43)	1.38 (1.33, 1.43)
16.0	1.40 (1.36, 1.44)	1.40 (1.34, 1.45)
17.0	1.42 (1.37, 1.46)	1.42 (1.36, 1.47)
18.0	1.43 (1.38, 1.48)	1.43 (1.37, 1.49)
19.0	1.44 (1.40, 1.49)	1.44 (1.38, 1.51)
20.0	1.46 (1.41, 1.51)	1.46 (1.39, 1.52)
21.0	1.47 (1.42, 1.52)	1.47 (1.40, 1.54)
22.0	1.48 (1.43, 1.53)	1.48 (1.41, 1.55)
23.0	1.49 (1.44, 1.54)	1.49 (1.42, 1.56)
24.0	1.50 (1.44, 1.55)	1.50 (1.43, 1.57)
25.0	1.51 (1.45, 1.56)	1.51 (1.43, 1.58)
26.0	1.52 (1.46, 1.57)	1.52 (1.44, 1.59)
27.0	1.52 (1.46, 1.58)	1.52 (1.45, 1.60)
28.0	1.53 (1.47, 1.59)	1.53 (1.45, 1.61)
29.0	1.54 (1.48, 1.60)	1.54 (1.46, 1.62)
30.0	1.55 (1.48, 1.61)	1.55 (1.46, 1.63)
31.0	1.55 (1.49, 1.61)	1.55 (1.47, 1.63)
32.0	1.56 (1.50, 1.62)	1.56 (1.48, 1.64)
33.0	1.57 (1.50, 1.63)	1.57 (1.48, 1.65)
34.0	1.57 (1.51, 1.64)	1.57 (1.49, 1.66)
35.0	1.58 (1.51, 1.64)	1.58 (1.49, 1.67)
36.0	1.58 (1.52, 1.65)	1.58 (1.50, 1.67)

37.0	1.59 (1.52, 1.65)	1.59 (1.50, 1.68)
38.0	1.59 (1.53, 1.66)	1.59 (1.50, 1.68)
39.0	1.60 (1.53, 1.66)	1.60 (1.51, 1.68)
40.0	1.60 (1.53, 1.66)	1.60 (1.51, 1.69)
41.0	1.60 (1.53, 1.66)	1.60 (1.51, 1.69)
42.0	1.60 (1.53, 1.67)	1.60 (1.51, 1.69)
43.0	1.60 (1.53, 1.67)	1.60 (1.51, 1.69)
44.0	1.60 (1.53, 1.67)	1.60 (1.51, 1.69)
45.0	1.60 (1.53, 1.67)	1.60 (1.51, 1.69)
46.0	1.60 (1.53, 1.67)	1.60 (1.51, 1.69)
47.0	1.60 (1.53, 1.67)	1.60 (1.51, 1.69)
48.0	1.60 (1.53, 1.67)	1.60 (1.51, 1.69)

Supplementary Table 9. Risk Estimates for $PM_{2.5}$ and VaD. Mean relative risk measures of VaD dementia risk across ambient $PM_{2.5}$ exposure Relative risk (RR) based on the mean relative risk function (along with the 95% uncertainty interval (UI) when not incorporating between-study heterogeneity and when accounting for between-study heterogeneity), presented at every 1 µg/m^3 of $PM_{2.5}$ from 1 to 40 µg/m^3. The mean RRs are calculated in comparison to a reference $PM_{2.5}$ level of 2.0 µg/m^3.

Ambient $PM_{2.5}$ level (µg/m^3)	RR (95% UI without between-study heterogeneity)	RR (95% UI with between-study heterogeneity)
2.0	1 (ref.)	1 (ref.)
3.0	1.19 (0.99, 1.45)	1.19 (0.97, 1.50)
4.0	1.39 (0.97, 1.98)	1.39 (0.94, 2.11)
5.0	1.58 (0.96, 2.60)	1.58 (0.92, 2.85)
6.0	1.78 (0.95, 3.30)	1.78 (0.90, 3.72)
7.0	1.97 (0.95, 4.08)	1.97 (0.89, 4.69)
8.0	2.15 (0.94, 4.92)	2.15 (0.87, 5.76)
9.0	2.33 (0.93, 5.79)	2.33 (0.86, 6.90)
10.0	2.50 (0.93, 6.68)	2.50 (0.85, 8.07)
11.0	2.65 (0.92, 7.56)	2.65 (0.84, 9.25)
12.0	2.79 (0.92, 8.40)	2.79 (0.83, 10.38)
13.0	2.91 (0.92, 9.16)	2.91 (0.83, 11.42)
14.0	3.01 (0.91, 9.84)	3.01 (0.82, 12.35)

15.0	3.09 (0.91, 10.41)	3.09 (0.82, 13.14)
16.0	3.16 (0.91, 10.88)	3.16 (0.81, 13.80)
17.0	3.21 (0.91, 11.26)	3.21 (0.81, 14.32)
18.0	3.25 (0.91, 11.55)	3.25 (0.81, 14.73)
19.0	3.28 (0.91, 11.76)	3.28 (0.81, 15.02)
20.0	3.30 (0.91, 11.90)	3.30 (0.81, 15.22)
21.0	3.31 (0.91, 11.98)	3.31 (0.81, 15.34)
22.0	3.31 (0.91, 12.03)	3.31 (0.81, 15.40)
23.0	3.32 (0.91, 12.05)	3.32 (0.81, 15.43)
24.0	3.32 (0.91, 12.05)	3.32 (0.81, 15.43)
25.0	3.32 (0.91, 12.05)	3.32 (0.81, 15.43)
26.0	3.32 (0.91, 12.05)	3.32 (0.81, 15.43)
27.0	3.32 (0.91, 12.05)	3.32 (0.81, 15.43)
28.0	3.32 (0.91, 12.05)	3.32 (0.81, 15.43)
29.0	3.32 (0.91, 12.05)	3.32 (0.81, 15.43)
30.0	3.32 (0.91, 12.05)	3.32 (0.81, 15.43)
31.0	3.32 (0.91, 12.05)	3.32 (0.81, 15.43)
32.0	3.32 (0.91, 12.05)	3.32 (0.81, 15.43)
33.0	3.32 (0.91, 12.05)	3.32 (0.81, 15.43)
34.0	3.32 (0.91, 12.05)	3.32 (0.81, 15.43)
35.0	3.32 (0.91, 12.05)	3.32 (0.81, 15.43)
36.0	3.32 (0.91, 12.05)	3.32 (0.81, 15.43)
37.0	3.32 (0.91, 12.05)	3.32 (0.81, 15.43)
38.0	3.32 (0.91, 12.05)	3.32 (0.81, 15.43)
39.0	3.32 (0.91, 12.05)	3.32 (0.81, 15.43)
40.0	3.32 (0.91, 12.05)	3.32 (0.81, 15.43)

Section 6: GATHER and PRISMA checklist

Supplementary Table 10. PRISMA 2020 checklist. From: Page MJ, McKenzie JE, Bossuyt PM, Boutron I, Hoffmann TC, Mulrow CD, et al. The PRISMA 2020 statement: an updated guideline for reporting systematic reviews. BMJ 2021;372:n71. doi: 10.1136/bmj.n71. For more information, visit: http://www.prisma-statement.org/.

Section and Topic	Item #	Checklist item	Location where item is reported
**TITLE**			
Title	1	Identify the report as a systematic review.	Yes, we included "systematic review" in the title.
**ABSTRACT**			
Abstract	2	See the PRISMA 2020 for Abstracts checklist.	See PRISMA 2020 for Abstracts Checklist below (Supplementary Table 11)
**INTRODUCTION**			
Rationale	3	Describe the rationale for the review in the context of existing knowledge.	See Introduction section paragraphs 1-3
Objectives	4	Provide an explicit statement of the objective(s) or question(s) the review addresses.	See Introduction section paragraph 5
**METHODS**			
Eligibility criteria	5	Specify the inclusion and exclusion criteria for the review and how studies were grouped for the syntheses.	Exclusion criteria summarized in Methods section "Step 1: Systematic literature review and data extraction" paragraphs 2 & 3; Full inclusion and exclusion criteria listed in SI Section 1 "Data source identification and assessment"; Reasons for exclusion and number of studies excluded also provided in PRISMA flow diagram (Extended Data Fig. 1)
Information sources	6	Specify all databases, registers, websites, organisations, reference lists and other sources searched or consulted to identify studies. Specify the date when each source was last searched or consulted.	Methods section "Step 1: Systematic literature review and data extraction" paragraphs 1; SI Section 1 "Data source identification and assessment"
Search strategy	7	Present the full search strategies for all databases, registers and websites, including any filters and limits used.	SI section 1.1 "Literature identification" (reference given at the end of paragraph 1 of "Step 1: Systematic literature review and data extraction" Methods section)
Selection process	8	Specify the methods used to decide whether a study met the inclusion criteria of the review, including how many reviewers screened each record and each report retrieved, whether they worked independently, and if applicable, details of automation tools used in the process.	Methods section "Step 1: Systematic literature review and data extraction" paragraph 1
Data collection	9	Specify the methods used to collect data from reports, including how many reviewers collected	Methods section "Step 1: Systematic

Section and Topic	Item #	Checklist item	Location where item is reported
process		data from each report, whether they worked independently, any processes for obtaining or confirming data from study investigators, and if applicable, details of automation tools used in the process.	literature review and data extraction"
Data items	10a	List and define all outcomes for which data were sought. Specify whether all results that were compatible with each outcome domain in each study were sought (e.g. for all measures, time points, analyses), and if not, the methods used to decide which results to collect.	Methods section "Step 1: Systematic literature review and data extraction" and SI sections 2 & 3
	10b	List and define all other variables for which data were sought (e.g. participant and intervention characteristics, funding sources). Describe any assumptions made about any missing or unclear information.	Methods section "Step 1: Systematic literature review and data extraction"; SI sections 2 "Study characteristics"; SI section 1 Supplementary Table 2 "Causal criteria extraction template" with definitions of all variables
Study risk of bias assessment	11	Specify the methods used to assess risk of bias in the included studies, including details of the tool(s) used, how many reviewers assessed each study and whether they worked independently, and if applicable, details of automation tools used in the process.	Methods section "Assess and adjust for biases"; SI section 3 "Study quality and risk of bias assessment"
Effect measures	12	Specify for each outcome the effect measure(s) (e.g. risk ratio, mean difference) used in the synthesis or presentation of results.	Main methods "overview", sections "Step 1: Systematic literature review and data extraction" and "Step 2: Estimate the risk–outcome relationships"
Synthesis methods	13a	Describe the processes used to decide which studies were eligible for each synthesis (e.g. tabulating the study intervention characteristics and comparing against the planned groups for each synthesis (item #5)).	Description of processes available in SI section 1 "Data source identification and assessment"
	13b	Describe any methods required to prepare the data for presentation or synthesis, such as handling of missing summary statistics, or data conversions.	Methods section "Step 1: Systematic literature review and data extraction"
	13c	Describe any methods used to tabulate or visually display results of individual studies and syntheses.	Methods section "Step 1: Systematic literature review and data extraction" and "Step 2: Estimate the risk–outcome relationships"
	13d	Describe any methods used to synthesize results and provide a rationale for the choice(s). If meta-analysis was performed, describe the model(s), method(s) to identify the presence and extent of statistical heterogeneity, and software package(s) used.	Methods sections Step 2 - Step4; software packages described in "Code availability"
	13e	Describe any methods used to explore possible causes of heterogeneity among study results (e.g.	Methods section "Step 4: Quantify in-between study heterogeneity and

Section and Topic	Item #	Checklist item	Location where item is reported
		subgroup analysis, meta-regression).	adjust for within study correlation”
	13f	Describe any sensitivity analyses conducted to assess robustness of the synthesized results.	Methods section “Step 2: Estimate the risk–outcome relationships”
Reporting bias assessment	14	Describe any methods used to assess risk of bias due to missing results in a synthesis (arising from reporting biases).	Methods section “Step 5: Evaluate risk of publication or reporting bias
Certainty assessment	15	Describe any methods used to assess certainty (or confidence) in the body of evidence for an outcome.	Methods section “Step 4: Quantify in-between study heterogeneity and adjust for within study correlation”
**RESULTS**			
Study selection	16a	Describe the results of the search and selection process, from the number of records identified in the search to the number of studies included in the review, ideally using a flow diagram.	PRISMA flow diagram Extended Data Figure 1
	16b	Cite studies that might appear to meet the inclusion criteria, but which were excluded, and explain why they were excluded.	Supplementary Table 4 “Full-text reviewed studies and inclusion/exclusion decisions”
Study characteristics	17	Cite each included study and present its characteristics.	Supplementary Table 5 “Study characteristics”
Risk of bias in studies	18	Present assessments of risk of bias for each included study.	Results section “Risk of bias assessment”; SI section 3 “Study quality and risk of bias assessment” (Supplementary Table 6)
Results of individual studies	19	For all outcomes, present, for each study: (a) summary statistics for each group (where appropriate) and (b) an effect estimate and its precision (e.g. confidence/credible interval), ideally using structured tables or plots.	Extended Data Fig. 2 forest plot; SI section 2 “Study characteristics” (Supplemental Table 5)
Results of syntheses	20a	For each synthesis, briefly summarise the characteristics and risk of bias among contributing studies.	Results section “$PM_{2.5}$ and Dementia Relationships” and “Risk of bias assessment”
	20b	Present results of all statistical syntheses conducted. If meta-analysis was done, present for each the summary estimate and its precision (e.g. confidence/credible interval) and measures of statistical heterogeneity. If comparing groups, describe the direction of the effect.	Results section “$PM_{2.5}$ and Dementia Relationships” and “Subgroup Analysis”; Figures 1-3; SI section 5 Supplementary Table 7-9.
	20c	Present results of all investigations of possible causes of heterogeneity among study results.	All 95% uncertainty intervals presented everywhere in the manuscript and SI reflect between-study heterogeneity (unless specified otherwise); BPRFs, ROSs, and star-ratings also reflect between-study heterogeneity
	20d	Present results of all sensitivity analyses conducted to assess the robustness of the synthesized results.	Results section “Sensitivity analysis” and SI section 4 “Sensitivity analysis”
Reporting	21	Present assessments of risk of bias due to	Results section “risk of bias

Section and Topic	Item #	Checklist item	Location where item is reported
biases		missing results (arising from reporting biases) for each synthesis assessed.	assessment”; funnel plots (Fig. 1b–3b); SI section 4 “Sensitivity analysis”
Certainty of evidence	22	Present assessments of certainty (or confidence) in the body of evidence for each outcome assessed.	All estimates are presented with 95% uncertainty intervals. UI values are given alongside all mean estimates in the Results and Discussion sections as well as in Supplementary Table 7-9.
**DISCUSSION**			
Discussion	23a	Provide a general interpretation of the results in the context of other evidence.	Discussion section paragraphs 1-4
	23b	Discuss any limitations of the evidence included in the review.	Discussion section paragraph 6
	23c	Discuss any limitations of the review processes used.	Discussion section paragraph 6
	23d	Discuss implications of the results for practice, policy, and future research.	Discussion section paragraph 7-8
**OTHER INFORMATION**			
Registration and protocol	24a	Provide registration information for the review, including register name and registration number, or state that the review was not registered.	Registered at PROSPERO (ID: CRD42023421869)
	24b	Indicate where the review protocol can be accessed, or state that a protocol was not prepared.	The protocol can be accessed through PROSPERO website: https://www.crd.york.ac.uk/prospero/
	24c	Describe and explain any amendments to information provided at registration or in the protocol.	No amendments
Support	25	Describe sources of financial or non-financial support for the review, and the role of the funders or sponsors in the review.	“Acknowledgments” section in the main text
Competing interests	26	Declare any competing interests of review authors.	“Competing interests” section in the main text
Availability of data, code and other materials	27	Report which of the following are publicly available and where they can be found: template data collection forms; data extracted from included studies; data used for all analyses; analytic code; any other materials used in the review.	“Data availability” and “Code availability” sections in the main text; data collection form template: Supplementary Table 2.

Supplementary Table 11. PRISMA 2020 abstract checklist. From: Page MJ, McKenzie JE, Bossuyt PM, Boutron I, Hoffmann TC, Mulrow CD, et al. The PRISMA 2020 statement: an updated guideline for reporting systematic reviews. BMJ 2021;372:n71. doi: 10.1136/bmj.n71. For more information, visit: http://www.prisma-statement.org/.

Section and Topic	Item #	Checklist item	Reported (Yes/No)
TITLE			
Title	1	Identify the report as a systematic review.	Yes, we included "systematic review" in the title.
BACKGROUND			
Objectives	2	Provide an explicit statement of the main objective(s) or question(s) the review addresses.	Yes
METHODS			
Eligibility criteria	3	Specify the inclusion and exclusion criteria for the review.	Not in abstract, just main text and SI Section 1.2 (given word count limitations by the journal)
Information sources	4	Specify the information sources (e.g. databases, registers) used to identify studies and the date when each was last searched.	Not in abstract, just main text and SI Section 1.1 (given word count limitations by the journal)
Risk of bias	5	Specify the methods used to assess risk of bias in the included studies.	Not in abstract, just main text and SI Section 3 (given word count limitations by the journal)
Synthesis of results	6	Specify the methods used to present and synthesize results.	Yes
RESULTS			
Included studies	7	Give the total number of included studies and participants and summaries relevant characteristics of studies.	Not in abstract, just main text and SI Table 5 (given word count limitations by the journal)
Synthesis of results	8	Present results for main outcomes, preferably indicating the number of included studies and participants for each. If meta-analysis was done, report the summary estimate and confidence/credible interval. If comparing groups, indicate the direction of the effect (i.e. which group is favored).	Yes, we included the number of data points included in the mate-analysis. The summary statistics, confidence intervals, and reference level are all included in the abstract. Detailed characteristic regarding included studies is only provided in the Supplementary Table 5. (given word count limitations by the journal)
DISCUSSION			
Limitations of evidence	9	Provide a brief summary of the limitations of the evidence included in the review (e.g. study risk of bias, inconsistency and imprecision).	Not in abstract, just main text discussion section (given word count limitations by the journal)
Interpretation	10	Provide a general interpretation of the results and important implications.	Yes
OTHER			
Funding	11	Specify the primary source of funding for the review.	Not in abstract, just Acknowledgement (given word count limitations by the journal)
Registration	12	Provide the register name and registration number.	Not in abstract, just main text (given word count limitations by the journal)

Supplementary Table 12. GATHER checklist. From: Stevens GA, Alkema L, Black RE, Boerma JT, Collins GS, Ezzati M, et al. Guidelines for accurate and transparent health estimates reporting: The GATHER statement. Lancet. 2016. doi: http://dx.doi.org/10.1016/S0140-6736(16)30388-9.

Item #	Checklist item	Reported on page #
**Objectives and funding**		
1	Define the indicator(s), populations (including age, sex, and geographic entities), and time period(s) for which estimates were made.	Methods section "Step 1: Systematic literature review and data extraction"
2	List the funding sources for the work.	Main text acknowledgement section
**Data Inputs**		
For all data inputs from multiple sources that are synthesized as part of the study:		
3	Describe how the data were identified and how the data were accessed.	Methods section "Step 1: Systematic literature review and data extraction" and SI section 1 "Data source identification and assessment"
4	Specify the inclusion and exclusion criteria. Identify all ad-hoc exclusions.	SI section 1.2 "Assessing data source eligibility"
5	Provide information on all included data sources and their main characteristics. For each data source used, report reference information or contact name/institution, population represented, data collection method, year(s) of data collection, sex and age range, diagnostic criteria or measurement method, and sample size, as relevant.	SI section 2 Supplementary Table 5 "study characteristics"
6	Identify and describe any categories of input data that have potentially important biases (e.g., based on characteristics listed in item 5).	Results section "risk of bias assessment"; details in SI section 3 and specifically, Supplementary Table 6 "study quality for every study used in the models"
For data inputs that contribute to the analysis but were not synthesized as part of the study:		
7	Describe and give sources for any other data inputs.	N/A
For all data inputs:		
8	Provide all data inputs in a file format from which data can be efficiently extracted (e.g., a spreadsheet rather than a PDF), including all relevant meta-data listed in item 5. For any data inputs that cannot be shared because of ethical or legal reasons, such as third-party ownership, provide a contact name or the name of the institution that retains the right tothe data.	"Data availability" section in the main text
**Data analysis**		
9	Provide a conceptual overview of the data analysis method. A diagram may be helpful.	Main text methods overview; PRISMA flow diagram (Extended Data Figure 1)
10	Provide a detailed description of all steps of the analysis, including mathematical formulae. This description should cover, as relevant, data cleaning, data pre-processing, data adjustments and weighting of data sources, and mathematical or statistical model(s).	Main text methods; See Zheng et al. for additional detail

11	Describe how candidate models were evaluated and how the final model(s) were selected.	Main text methods section
12	Provide the results of an evaluation of model performance, if done, as well as the results of any relevant sensitivity analysis.	Main text methods section
13	Describe methods for calculating uncertainty of the estimates. State which sources of uncertainty were, and were not, accounted for in the uncertainty analysis.	Methods section "Step 4: Quantify in-between study heterogeneity and adjust for within study correlation"
14	State how analytic or statistical source code used to generate estimates can be accessed.	"Code availability" section in the main text
**Results and Discussion**		
15	Provide published estimates in a file format from which data can be efficiently extracted.	N/A
16	Report a quantitative measure of the uncertainty of the estimates (e.g. uncertainty intervals).	All estimates are presented with 95% uncertainty intervals. UI values are given alongside all mean estimates in the Results and Discussion sections as well as in Supplementary Table 7-9.
17	Interpret results in light of existing evidence. If updating a previous set of estimates, describe the reasons for changes in estimates.	Discussion section
18	Discuss limitations of the estimates. Include a discussion of any modelling assumptions or data limitations that affect interpretation of the estimates.	Discussion section paragraph 6
