# Supplementary material for: A systematic review with a Burden of Proof meta-analysis of health effects of long-term ambient fine particulate matter (PM2.5) exposure on dementia
Source: Nat Aging. 2025 Mar 21;5(5):897–908. doi: 10.1038/s43587-025-00844-y (PMC12092285; doi:10.1038/s43587-025-00844-y)
Supplement: Supplementary file 2 — Reporting Summary [file 43587_2025_844_MOESM2_ESM.pdf]

nature portfolio

Corresponding author(s): Katrin Burkart

Last updated by author(s): Feb 1, 2025

# Reporting Summary

Nature Portfolio wishes to improve the reproducibility of the work that we publish. This form provides structure for consistency and transparency in reporting. For further information on Nature Portfolio policies, see our Editorial Policies and the Editorial Policy Checklist.

## Statistics

For all statistical analyses, confirm that the following items are present in the figure legend, table legend, main text, or Methods section.

| n/a | Confirmed | |
|---|---|---|
| ☐ | ☒ | The exact sample size (*n*) for each experimental group/condition, given as a discrete number and unit of measurement |
| ☐ | ☒ | A statement on whether measurements were taken from distinct samples or whether the same sample was measured repeatedly |
| ☒ | ☐ | The statistical test(s) used AND whether they are one- or two-sided<br>*Only common tests should be described solely by name; describe more complex techniques in the Methods section.* |
| ☐ | ☒ | A description of all covariates tested |
| ☐ | ☒ | A description of any assumptions or corrections, such as tests of normality and adjustment for multiple comparisons |
| ☐ | ☒ | A full description of the statistical parameters including central tendency (e.g. means) or other basic estimates (e.g. regression coefficient) AND variation (e.g. standard deviation) or associated estimates of uncertainty (e.g. confidence intervals) |
| ☐ | ☒ | For null hypothesis testing, the test statistic (e.g. *F*, *t*, *r*) with confidence intervals, effect sizes, degrees of freedom and *P* value noted<br>*Give P values as exact values whenever suitable.* |
| ☐ | ☒ | For Bayesian analysis, information on the choice of priors and Markov chain Monte Carlo settings |
| ☒ | ☐ | For hierarchical and complex designs, identification of the appropriate level for tests and full reporting of outcomes |
| ☒ | ☐ | Estimates of effect sizes (e.g. Cohen's *d*, Pearson's *r*), indicating how they were calculated |

*Our web collection on statistics for biologists contains articles on many of the points above.*

## Software and code

Policy information about availability of computer code

| | |
|---|---|
| Data collection | No data collection was carried out for this analysis |
| Data analysis | Analyses were carried out using R version 4.0.5 and Python version 3.10.9. All code used for these analyses is publicly available online (https://github.com/ihmeuw-msca/burden-of-proof/). |

For manuscripts utilizing custom algorithms or software that are central to the research but not yet described in published literature, software must be made available to editors and reviewers. We strongly encourage code deposition in a community repository (e.g. GitHub). See the Nature Portfolio guidelines for submitting code & software for further information.

## Data

Policy information about availability of data

All manuscripts must include a data availability statement. This statement should provide the following information, where applicable:

- Accession codes, unique identifiers, or web links for publicly available datasets
- A description of any restrictions on data availability
- For clinical datasets or third party data, please ensure that the statement adheres to our policy

The findings of this study are based on data extracted from published literature up to June 2023. We conducted systematic literature search in PubMed (https://pubmed.ncbi.nlm.nih.gov), Embase (https://www.embase.com), and Web of Science (https://www.webofscience.com), with detailed search terms provided in Supplementary Information Section 1.1.2. We have included the full reference list for all studies incorporated in the systematic review and meta-regression.



April 2023

Supplementary Table 4 presents the full-text reviewed studies, along with the detailed rationale for inclusion or exclusion in the meta-regression. For studies included in the meta-regression, study characteristics and detailed extracted effect sizes are presented, Supplementary Table 5 and Table 6, and Extended Data Fig. 2. The template of the data collection form is provided in Supplementary Information Section 1 (Supplementary Table 2).

## Research involving human participants, their data, or biological material

Policy information about studies with human participants or human data. See also policy information about sex, gender (identity/presentation), and sexual orientation and race, ethnicity and racism.

| | |
|---|---|
| Reporting on sex and gender | No primary data collection was carried out for this analysis, so the study does not involve human research participants. As stated in the methods overview, our estimates are not specific to or disaggregated by sex. Because of this, we included all available data regardless of how or if the input study collected and reported data by sex or gender. We did not perform sex- or gender-based analyses due to limitations in and scarcity of the underlying data. |
| Reporting on race, ethnicity, or other socially relevant groupings | No primary data collection was carried out for this analysis, so the study does not involve human research participants. As stated in the methods overview, our estimates are not specific to or disaggregated by race, ethnicity or other socially relevant groupings. Because of this, we included all available data regardless of how or if the input study collected. We did not perform race/ethnicity-based analyses due to limitations in and scarcity of the underlying data. |
| Population characteristics | No primary data collection was carried out for this analysis, so the study does not involve human research participants. The analysis evaluated the effect of long-term PM2.5 exposure on dementia among adults aged 30 years and above. |
| Recruitment | No primary data collection was carried out for this analysis, so we did not recruit participants. |
| Ethics oversight | This study was approved by the University of Washington IRB Committee. |

Note that full information on the approval of the study protocol must also be provided in the manuscript.

# Field-specific reporting

Please select the one below that is the best fit for your research. If you are not sure, read the appropriate sections before making your selection.

☒ Life sciences ☐ Behavioural & social sciences ☐ Ecological, evolutionary & environmental sciences

For a reference copy of the document with all sections, see nature.com/documents/nr-reporting-summary-flat.pdf

# Life sciences study design

All studies must disclose on these points even when the disclosure is negative.

| | |
|---|---|
| Sample size | Our meta-analysis included all available evidence from longitudinal cohort studies up to June 2023. Following literature selection based on our inclusion/exclusion criteria (Supplementary Information Section 1.2), we included 49 effect estimates from 28 longitudinal epidemiological studies examining the association between PM2.5 and dementia. A PRISMA flowchart (Extended Data Fig. 1) provides detailed information on study selection and sample size. No power calculation was performed, as meta-analysis sample sizes are determined by available studies. The number of effect estimates was sufficient to ensure convergence of the meta-regression model. For each included cohort study, the original participant sample sizes are summarized in Supplementary Table 5. |
| Data exclusions | As described in Supplementary Information Section 1.2, reports were excluded based on the following exclusion criteria: study design (not cohort study designs), not population of interest (not adults aged 30 years and above), undesired exposure definition (not long-term PM2.5 exposure), no report of relative measure of risk, minimum required data not present, and duplicate study. |
| Replication | This study is a meta-analysis of existing longitudinal cohort studies on PM2.5 and dementia published up to June 2023. No new experiments or data collection were conducted. Therefore, traditional replication is not directly applicable. However, we have provided all data sources, selection criteria, and open-source code for statistical analysis, allowing other researchers to reproduce our findings using the same methodology. |
| Randomization | This analysis is a meta-analysis of existing studies and thus, there were no experimental groups. |
| Blinding | This study is a meta-analysis of existing longitudinal cohort studies; therefore, no blinding procedures were involved. |

# Reporting for specific materials, systems and methods

We require information from authors about some types of materials, experimental systems and methods used in many studies. Here, indicate whether each material, system or method listed is relevant to your study. If you are not sure if a list item applies to your research, read the appropriate section before selecting a response.

## Materials & experimental systems

| n/a | Involved in the study |
| --- | --- |
| ☒ | ☐ Antibodies |
| ☒ | ☐ Eukaryotic cell lines |
| ☒ | ☐ Palaeontology and archaeology |
| ☒ | ☐ Animals and other organisms |
| ☒ | ☐ Clinical data |
| ☒ | ☐ Dual use research of concern |
| ☒ | ☐ Plants |

## Methods

| n/a | Involved in the study |
| --- | --- |
| ☒ | ☐ ChIP-seq |
| ☒ | ☐ Flow cytometry |
| ☒ | ☐ MRI-based neuroimaging |

# Plants

| | |
| --- | --- |
| Seed stocks | We did not use any plant data. |
| Novel plant genotypes | We did not use any plant data. |
| Authentication | We did not use any plant data. |
